# Identification of Small Open Reading Frame-encoded Proteins in the Human Genome

**DOI:** 10.1093/gpbjnl/qzaf004

**Published:** 2025-02-07

**Authors:** Hitesh Kore, Satomi Okano, Keshava K Datta, Jackson Thorp, Parthiban Periasamy, Mayur Divate, Upekha Liyanage, Gunter Hartel, Shivashankar H Nagaraj, Harsha Gowda

**Affiliations:** Centre for Genomics and Personalised Health, Queensland University of Technology, Brisbane 4059, Australia; Cancer Precision Medicine Group, QIMR Berghofer Medical Research Institute, Brisbane 4006, Australia; Statistics Unit, QIMR Berghofer Medical Research Institute, Brisbane 4006, Australia; Proteomics and Metabolomics Platform, La Trobe University, Melbourne 3083, Australia; Translational Neurogenomics, QIMR Berghofer Medical Research Institute, Brisbane 4006, Australia; Cancer Precision Medicine Group, QIMR Berghofer Medical Research Institute, Brisbane 4006, Australia; Faculty of Medicine, The University of Queensland, Brisbane 4072, Australia; Institute of Molecular and Cell Biology (IMCB), Agency for Science, Technology and Research, Singapore 138673, Singapore; Centre for Genomics and Personalised Health, Queensland University of Technology, Brisbane 4059, Australia; Cancer and Population Studies Group, QIMR Berghofer Medical Research Institute, Brisbane 4006, Australia; Statistics Unit, QIMR Berghofer Medical Research Institute, Brisbane 4006, Australia; Centre for Genomics and Personalised Health, Queensland University of Technology, Brisbane 4059, Australia; Centre for Genomics and Personalised Health, Queensland University of Technology, Brisbane 4059, Australia; Cancer Precision Medicine Group, QIMR Berghofer Medical Research Institute, Brisbane 4006, Australia; Faculty of Medicine, The University of Queensland, Brisbane 4072, Australia

**Keywords:** Non-coding RNA, Protein-coding potential, Novel protein, sORF, SEP

## Abstract

One of the main goals of the Human Genome Project is to identify all protein-coding genes. There are ∼ 20,500 protein-coding genes annotated in the human reference databases. However, in the last few years, proteogenomics studies have predicted thousands of novel protein-coding regions, including low-molecular-weight proteins encoded by small open reading frames (sORFs) in untranslated regions of messenger RNAs and non-coding RNAs. Most of these predictions are based on bioinformatics analyses and ribosome footprint data. The validity of some of these sORF-encoded proteins (SEPs) has been established through functional characterization. With the growing number of predicted novel proteins, a strategy to identify reliable candidates that warrant further studies is needed. In this study, we developed an integrated proteogenomics workflow to identify a reliable set of novel protein-coding regions in the human genome based on their recurrent observations across multiple samples. Publicly available ribosome profiling and global proteomic datasets were used to establish protein-coding evidence. We predicted protein translation from 4008 sORFs based on recurrent ribosome occupancy signals across samples. In addition, we identified 825 SEPs based on proteomic data. Some of the novel protein-coding regions identified were located in genome-wide association study (GWAS) loci associated with various traits and disease phenotypes. Peptides from SEPs are also presented by major histocompatibility complex class I (MHC-I), similar to canonical proteins. Novel protein-coding regions reported in this study expand the current catalog of protein-coding genes and warrant experimental studies to elucidate their cellular functions and potential roles in human diseases.

## Graphical Abstract

**Graphical Abstract:**
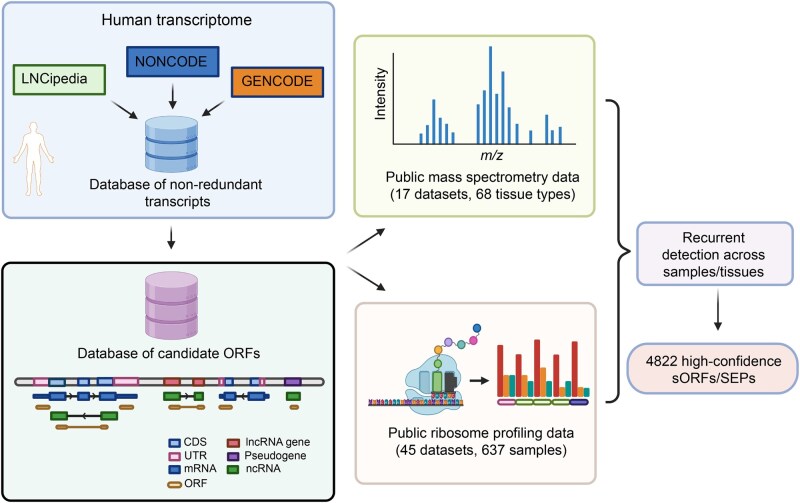

## Introduction

One of the primary goals of the Human Genome Project is to catalog all protein-coding genes. The draft map of the human genome was published in 2001 with a protein-coding gene set of 26,000–31,000 genes [1,2]. Since then, manual annotation teams at both National Center for Biotechnology Information (NCBI) and Ensembl have curated the list to arrive at ∼ 20,500 protein-coding genes. This curated gene list is used as a reference set by biologists and biomedical researchers. All the reagents including exon capture kits and antibodies are developed against this reference gene set. Any gene not represented in this reference gene set is less likely to be investigated by researchers.

Advances in DNA sequencing technologies have revolutionized our ability to sequence genomes and transcriptomes. Transcriptome sequencing studies in the past decade have revealed that most of the human genome is transcribed [3]. The advent of ribosome profiling (Ribo-seq) studies has shown ribosome occupancy on non-coding regions in addition to messenger RNAs (mRNAs) [4–9], including non-coding RNAs (ncRNAs) and untranslated regions (UTRs) in mRNAs. These observations have led to the prediction of several small open reading frame (sORF)-encoded proteins (SEPs) based on ribosome occupancy signals. This is also corroborated by proteomics studies [10,11]. SEPs play important roles in various biological processes, including mitochondrial and muscle function, DNA repair, development, and apoptosis [12–19]. However, the functions of the majority of SEPs remain unknown. Recent studies have also demonstrated these novel SEPs as a source of neoantigens presented on the surface of cancer cells [5,20]. As most of our understanding of human biology is predicated on knowing protein-coding gene repertoire and their function, cataloging all protein-coding genes in the human genome is vital. It is unclear how many protein-coding regions remain unannotated in the human genome.

Bioinformatics studies and high-throughput Ribo-seq studies have predicted thousands of novel protein-coding regions in the human genome [4,9,21,22]. These predicted candidates are available in public databases, including OpenProt, SmProt, and sORFs.org [23–25]. OpenProt, SmProt, and sORFs.org have cataloged over 400,000 [including > 38,000 ncRNA open reading frames (ORFs) with either Ribo-seq or proteomic evidence], 300,000, and 500,000 novel ORFs, respectively. The large numbers and high variability across different data resources raise concerns about the reliability of these predicted candidates and chance of many false positives. SEPs can be products of pervasive translation and a subset of them could be nonfunctional proteins [26]. It must be noted that mere detection of ORFs/SEPs is not proxy for their function and these candidates require systematic functional validations. Therefore, reference databases such as RefSeq, GENCODE, and UniProt are stringent to include these annotations as there is a risk of populating public databases used by global scientific community with several false positive entries. Therefore, there is a need for a strategy to distinguish sORFs that potentially code for proteins from those that may not. In a recent editorial, an international consortium has discussed the importance of annotating these novel proteins in public reference databases and the need for a strategy to identify reliable candidates [27]. In this study, we employed an integrated workflow to identify novel protein-coding regions that are supported by recurrent observations across multiple samples/datasets. These candidates can potentially serve as a reference set for functional studies aimed at investigating their roles in various biological processes and human diseases.

## Results

### Long ncRNAs have fewer exons and shorter ORFs

We calculated the exon count per transcript. Unlike mRNAs, most long ncRNAs (lncRNAs) have fewer exons (Figure S1A) [28]. Their conceptual translation revealed that the longest predicted ORF in most lncRNAs is shorter than 300 nt (Figure S1B). In addition, predicted ORFs in lncRNAs are poorly conserved across vertebrates compared to canonical ORFs in mRNAs (Figures S1C, S1D, and S2A). Since 2014, Ensembl has reclassified 66 lncRNAs as mRNAs that encode proteins (Table S1). They show moderate to high conservation across vertebrates. Shorter ORF length and/or low conservation may have excluded many of these proteins from initial genome annotation (Figure S2B) [22,29].

### Workflow for the identification of novel protein-coding regions in the human genome

**Figure 1:**
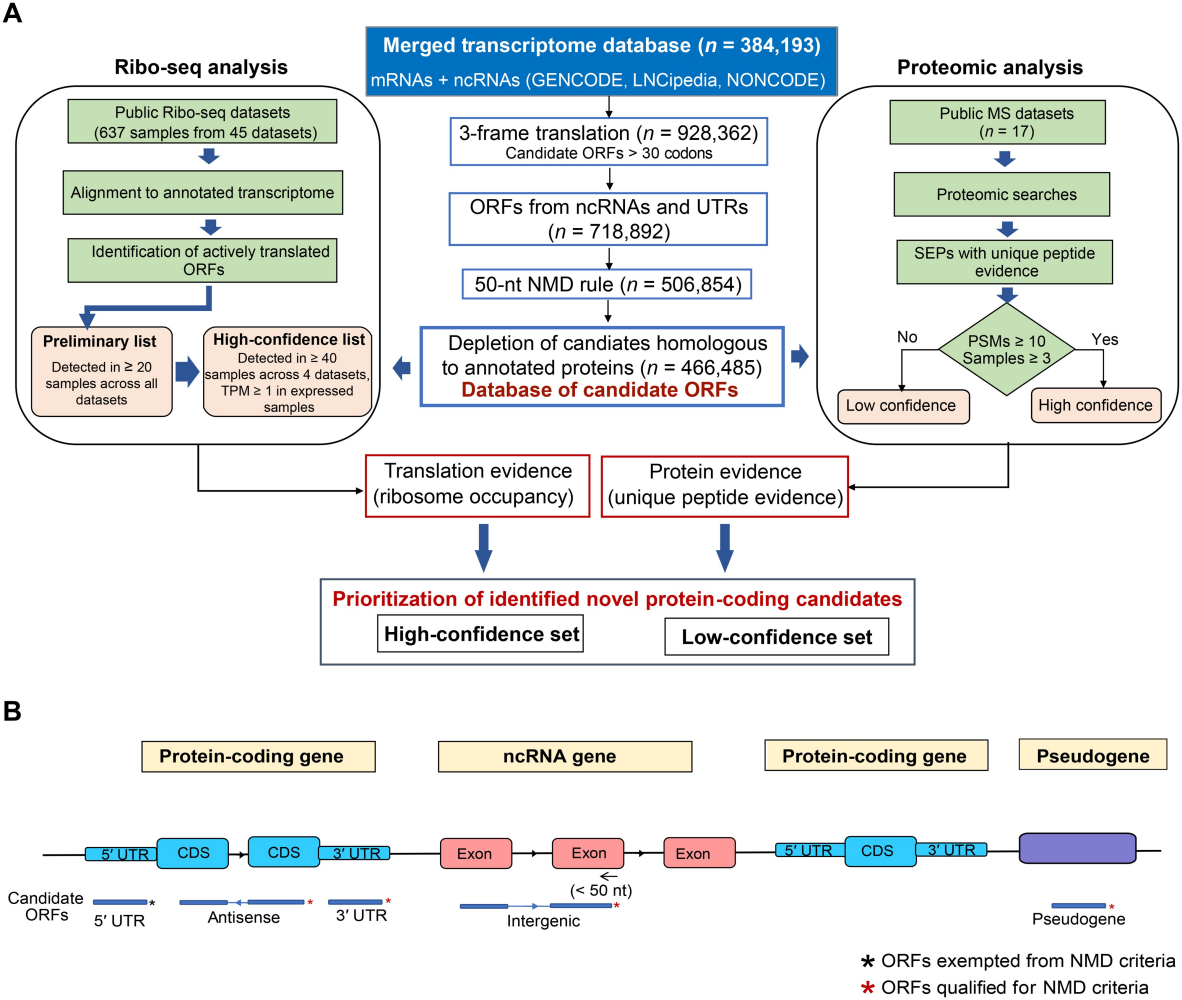
Strategy employed to identify novel protein-coding regions in the human genome **A**. Workflow employed to identify novel protein-coding regions in the human genome. **B**. Graphical representation of potential sources of novel ORFs in the human genome. ORF, open reading frame; mRNA, messenger RNA; ncRNA, non-coding RNA; TPM, transcripts per million; sORF, small open reading frame; PSM, peptide–spectrum match; SEP, small open reading frame-encoded protein; CDS, coding sequence; UTR, untranslated region; NMD, nonsense mediated decay; Ribo-seq, ribosome profiling sequencing; MS, mass spectrometry.

Unlike mRNAs, curation and annotation of ncRNAs are not consistent and streamlined. Different data resources catalog these transcripts using different approaches. In order to generate a comprehensive list of ncRNAs, we built a custom database by merging transcripts in GENCODE, LNCipedia, and NONCODE (Table S2). All the transcripts in the merged transcriptome database were translated in three reading frames, and ORFs > 30 codons were retained. Our approach for identifying novel protein-coding regions is summarized in **Figure 1**A and B. While the use of noncanonical start codons has been documented in the literature, the mechanisms underlying such alternative start site selection are poorly understood [30–32]. Therefore, we considered only those ORFs with an AUG start site. In addition, we removed transcripts that are potential targets of nonsense mediated decay (NMD). RefSeq and Ensembl annotation pipelines use similar criteria to classify transcripts with premature termination codons. We employed the 50-nt NMD rule in our workflow. ORFs with stop codons either in the last exon or less than 50 nt from the 3′ end of penultimate exon were considered. ORFs in 5′ UTRs of canonical mRNAs were exceptions to this rule. ORF biotypes were assigned based on their genomic position and transcript annotations (Table S3). Evolutionary conservation within these ORFs was determined based on conservation track generated for 100 vertebrates using the phyloP method [33]. We determined the cutoff for conservation based on conservation pattern in coding sequence (CDS) region of canonical genes. This enabled the development of a custom database with candidate ORFs which we refer to as ORFome database. It comprised 466,485 predicted ORFs including 34,562 that showed conservation in vertebrates (Table S3). Using this database, we analyzed publicly available Ribo-seq datasets (45 datasets comprising 637 samples) and global proteomic datasets (17 datasets comprising 392 samples from 68 different tissues/cell types). These ORFs were further classified into high- and low-confidence candidates based on their detection across multiple samples or datasets (Figure 1A).

### Protein translation evidence based on ribosome occupancy

**Figure 2:**
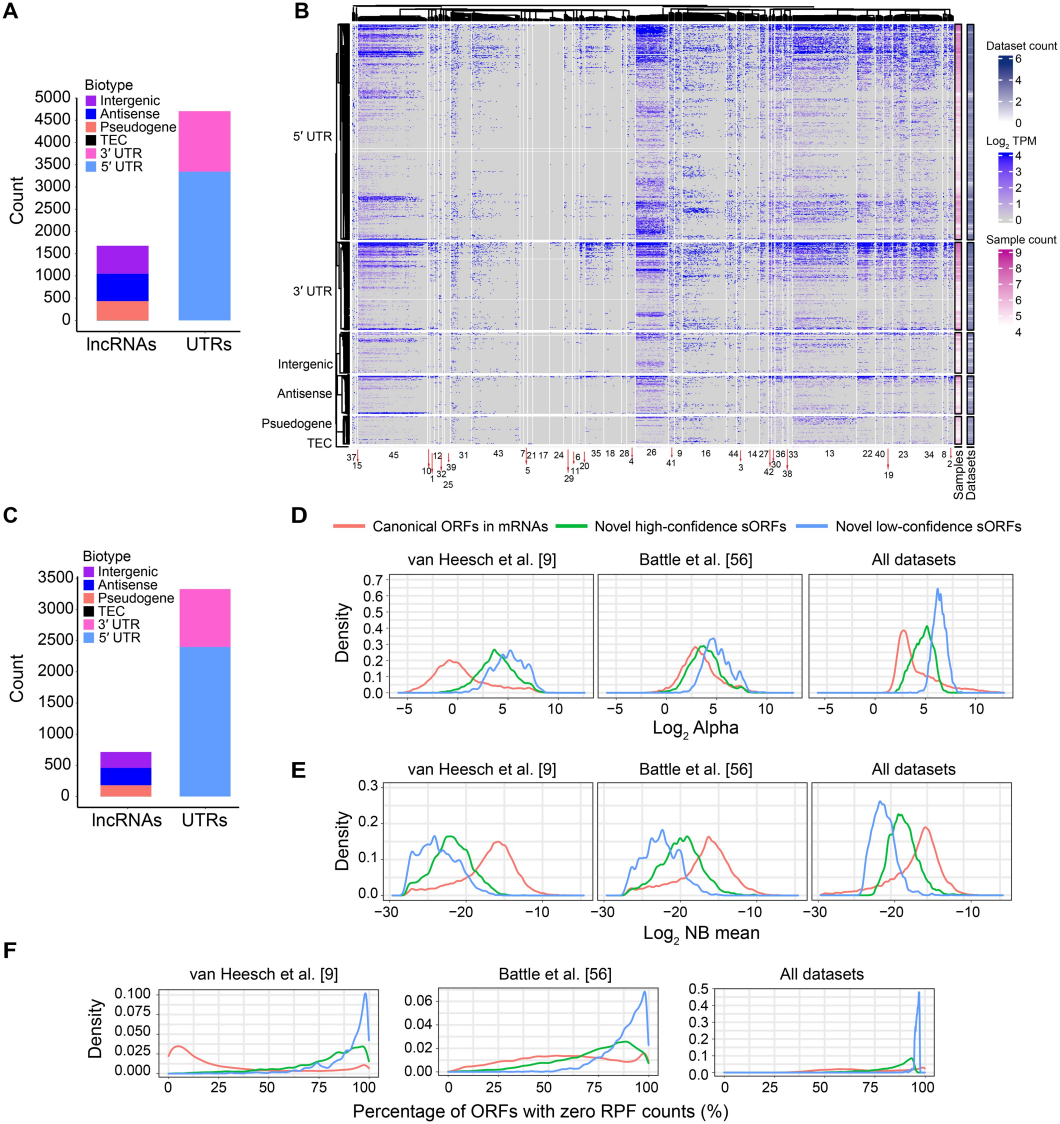
Novel protein-coding regions identified based on ribosome occupancy **A**. Number of sORFs with ribosome occupancy signals and their corresponding biotypes. These comprised a total of 6356 novel sORFs including 2348 low-confidence sORFs and 4008 high-confidence sORFs. **B**. Heatmap of sORFs with ribosome occupancy signals across datasets. Each column represents a dataset and rows represent sORFs (dataset index is provided in Table S4). The heatmap included a total of 6356 sORFs including 428 pseudogene, 591 antisense lncRNA, 629 intergenic lncRNA, 5 TEC, 1359 3′ UTR, and 3344 5′ UTR sORFs. **C**. Number of high-confidence sORFs and their corresponding biotypes. **D**. Distribution of the dispersion parameter alpha of RPFs in predicted sORFs compared to canonical ORFs in mRNAs. **E**. Distribution of the NB mean of RPFs in predicted sORFs compared to canonical ORFs in mRNAs. **F**. Proportion of ORFs with zero RPF counts across samples. lncRNA, long non-coding RNA; TEC, to be experimentally confirmed; RPF, ribosome-protected fragment; NB, negative binomial.

We identified ribosome occupancy signals in 50,610 candidate sORFs (Table S4). There were 6356 candidate sORFs that showed ribosome occupancy signals in at least 20 samples (Figure S3A). This included 629 intergenic lncRNA, 591 antisense lncRNA, 4703 UTR (1359 3′ UTR and 3344 5′ UTR), 428 pseudogene, and 5 to be experimentally confirmed (TEC) sORFs (**Figure 2**A). Figure 2B shows a heatmap of ribosome footprints among different biotypes across datasets. Previous studies have reported stochastic nature of ribosome occupancy footprints that can result in false positives [34]. In order to minimize false positives based on stochastic signals, we identified sORFs with ribosome occupancy signals in at least 40 samples across 4 independent datasets with median ribosome-protected fragment (RPF) abundance of ≥ 1 transcript per million (TPM) (Figure S3B). This resulted in 4008 candidate sORFs with translation evidence including 256 intergenic lncRNA, 3320 UTR, 256 antisense lncRNA, 175 pseudogene, and 1 TEC sORFs (Figure 2C).

We employed a negative binomial (NB) model to see if there is any difference in RPF signals in novel sORFs with higher recurrent detection compared to those detected in fewer samples. Genuine sORFs were expected to exhibit lower variation in RPF density from their average RPF abundance and a higher NB mean across samples. As expected, candidate sORFs meeting a higher evidence threshold for recurrent detection and RPF abundance showed lower dispersion (Figure 2D). Some candidates had dispersion values comparable to ORFs that encode canonical proteins. These candidates also showed higher NB means similar to ORFs that encode canonical proteins (Figure 2E). A higher NB mean corresponds to a higher proportion of samples with non-zero RPF counts for a given ORF in the dataset (Figure 2F). Overall, these results indicate that recurrent detection of RPF signals across multiple samples is a useful measure to identify reliable candidates and limits false positives that might result from stochastic RPF signals observed in few samples. The RPF abundance in ORF regions of transcripts encoding high-confidence candidate proteins was comparable to that of canonical ORFs in mRNAs (Figure S4A–C). We identified 370 previously reported sORFs, confirming the validity of our candidate list. Representative examples of novel sORFs are shown in Figure S5.

### Identification of SEPs from global proteomic datasets of various human tissues

**Figure 3:**
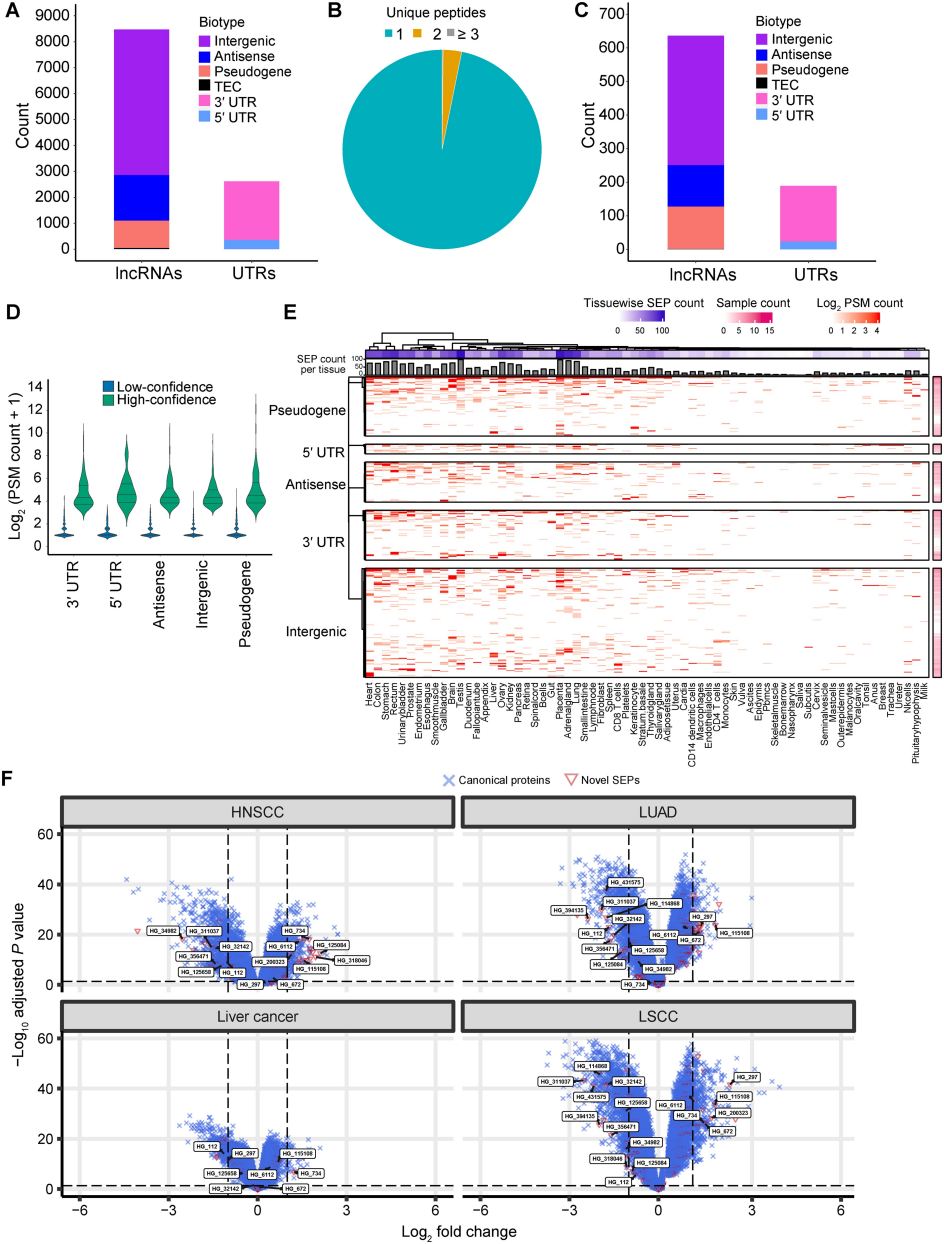
Novel SEPs identified based on proteomic evidence **A**. Number of SEPs with proteomic evidence and associated biotypes. **B**. Pie chart showing relative fraction of SEPs identified with 1, 2 and ≥ 3 unique peptides. **C**. Number of high-confidence SEPs and associated biotypes. **D**. Number of PSMs supporting high-confidence and low-confidence SEPs. **E**. Heatmap showing SEPs detected across different human tissues. Columns represent different tissues and rows represent SEPs. This heatmap included 12, 62, 50, 136, and 74 SEPs derived from 5′ UTRs, 3′ UTRs, antisense lncRNAs, intergenic lncRNAs, and pseudogenes, respectively. **F**. Volcano plots showing differentially expressed SEPs in HNSCC, LUAD, liver cancer, and LSCC. LUAD, lung adenocarcinoma; LSCC, lung squamous cell carcinoma; HNSCC, head and neck squamous cell carcinoma.

Proteomics data provide direct evidence of proteins unlike Ribo-seq. We searched publicly available proteomic datasets from multiple healthy and cancerous tissues to identify SEPs (Table S5). We identified peptides from 11,085 SEPs, including 5605 intergenic lncRNA, 1756 antisense lncRNA, 1064 pseudogene, 43 TEC, and 2617 UTR SEPs (**Figure 3**A). Due to the short length of most of these SEPs, the majority of them were supported by single peptide evidence (Figure 3B).

Candidates with ≥ 10 peptide–spectrum matches (PSMs) and detected in at least 3 samples were classified as high-confidence SEPs. Using this criterion, we narrowed down our candidate list to 825 high-confidence SEPs, including 385 intergenic lncRNA, 123 antisense lncRNA, 127 pseudogene, 1 TEC, and 189 UTR SEPs (Figure 3C). Figure 3D shows their PSM counts across biotypes. Many SEPs were identified from multiple tissues, while some showed tissue-restricted expression patterns (Figure 3E). Representative examples of observed and predicted tandem mass spectrometry (MS/MS) spectra from Prosit [35] confirmed the validity of these spectral assignments (Figure S6). Interestingly, 18 candidates from our list have also been reported in published studies (Table S6). In order to evaluate if expression of these SEPs is modulated in disease context, we carried out differential expression analysis using the Clinical Proteomic Tumor Analysis Consortium (CPTAC) datasets from four different cancers. This analysis identified a total of 82 differentially expressed SEPs, including 31 SEPs in head and neck squamous cell carcinoma (HNSCC), 28 in lung squamous cell carcinoma (LSCC), 32 in lung adenocarcinoma (LUAD), and 15 in liver cancer (Figure 3F). Among these, 17 SEPs showed differential expression patterns in more than one cancer type (Table S5; File S1), with 9 consistently down-regulated and 5 consistently up-regulated across cancer types. These candidates warrant functional validation to investigate their roles in these cancer types.

### Properties and characteristic features of sORFs

Transcripts that contain high-confidence sORFs were expressed at higher abundance than those containing low-confidence sORFs. Expression abundance of high-confidence candidates was comparable to that of mRNAs (Figure S7).

**Figure 4:**
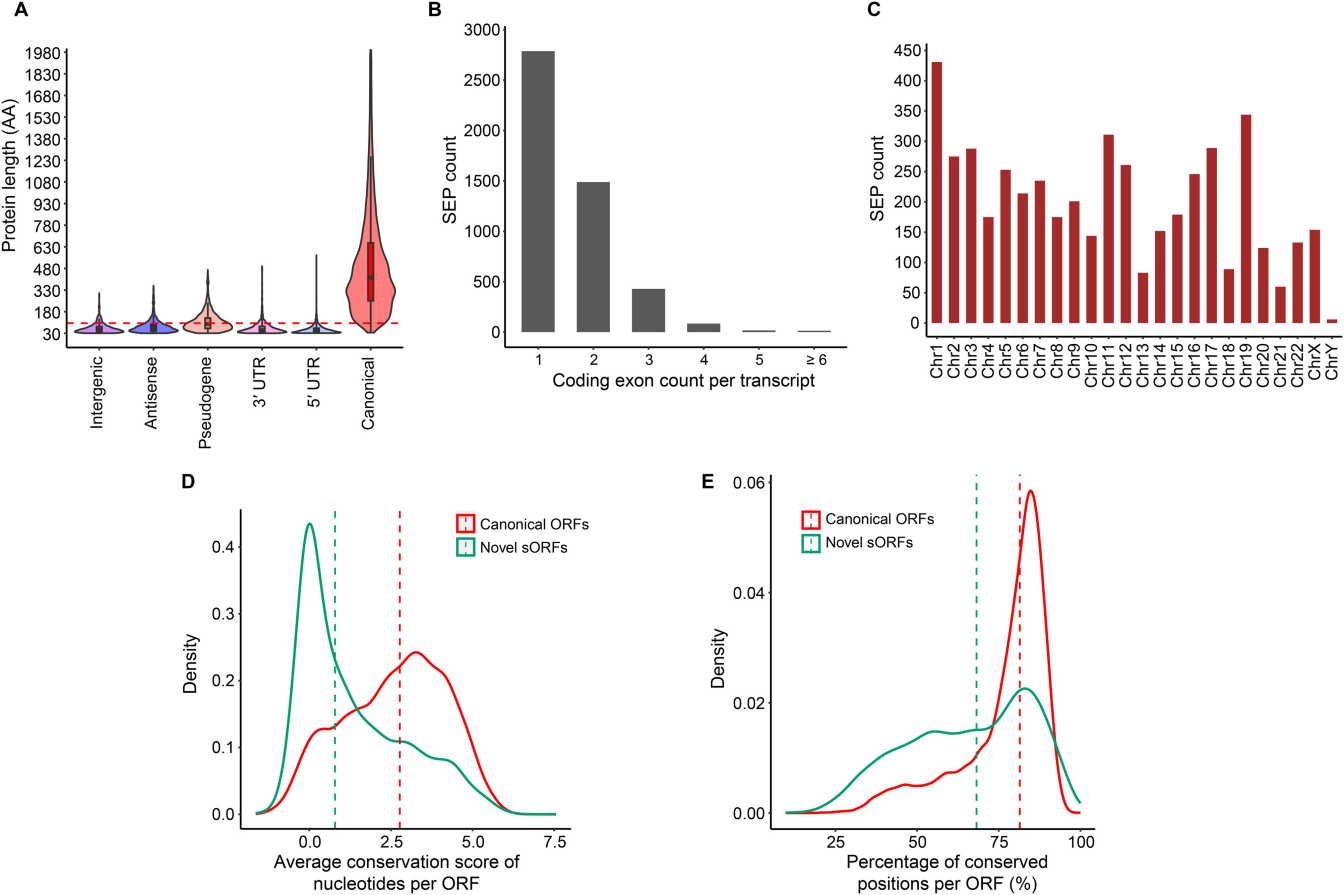
Characteristic features associated with high-confidence sORFs **A**. Length distribution of SEPs compared to canonical proteins. **B**. Distribution of SEPs encoded by transcripts with different numbers of coding exons. **C**. Number of SEPs encoded by different chromosomes. **D**. Distribution of average conservation scores of nucleotides for novel sORFs and canonical ORFs in mRNAs. Per-ORF conservation score was plotted for novel sORFs and canonical ORFs in mRNAs. **E**. Percentage of conserved nucleotides for predicted novel sORFs and canonical ORFs in mRNAs. AA, amino acid.

Combining high-confidence candidates based on ribosome occupancy and proteomic data resulted in the identification of 4822 sORFs with reliable evidence of protein-coding potential. Among these, 4232 sORFs encoded SEPs which were shorter than 100 amino acids (**Figure 4**A). Most protein-coding sORF candidates were in single-exon transcripts (Figure 4B). Although they were distributed across all chromosomes, chromosomes 1 and 19 contained most candidates (Figure 4C). While most sORFs are poorly conserved across vertebrates, a subset of sORFs (*n* = 1626) showed comparable conservation scores to those of canonical ORFs (Figure 4D). Although most nucleotides in canonical ORFs were conserved across vertebrates, only a fraction of sORFs showed this pattern (Figure 4E). The majority of conserved sORFs (*n* = 1270) could be explained by their overlap with annotated CDSs.

### Domain prediction in SEPs

We evaluated domain signatures in SEPs using InterProScan and identified 361 SEPs harboring domain signatures. Among these, 167 SEPs encoded by pseudogene sORFs harbored a higher number of domain signatures. However, 15 SEPs encoded by intergenic lncRNA sORFs also had conserved protein domains (Figure S8A). Two representative examples of SEPs with domain signatures are shown in Figure S8B. Subcellular localization prediction revealed 1001 SEPs that were potentially secreted, 26 with transmembrane domains (DeepTMHMM prediction), and 286 that may localize to mitochondria (TargetP-2.0 prediction) (Table S6).

Recent studies have shown that noncanonical SEPs have more disordered regions and may not form stable protein structures [21]. Several SEPs that are intrinsically disordered have been shown to play important roles in signaling, transcription, and translation processes [36]. We used IUPred3 to predict intrinsically disordered regions (IDRs) [37]. The residues with IUPred3 score ≥ 0.5 were considered disordered, and the fraction of disordered residues for each SEP was calculated. There were 1350 SEPs with at least 50% disordered residue content (Table S6).

### Mutations and GWAS variants in SEP-encoding genomic regions

**Figure 5:**
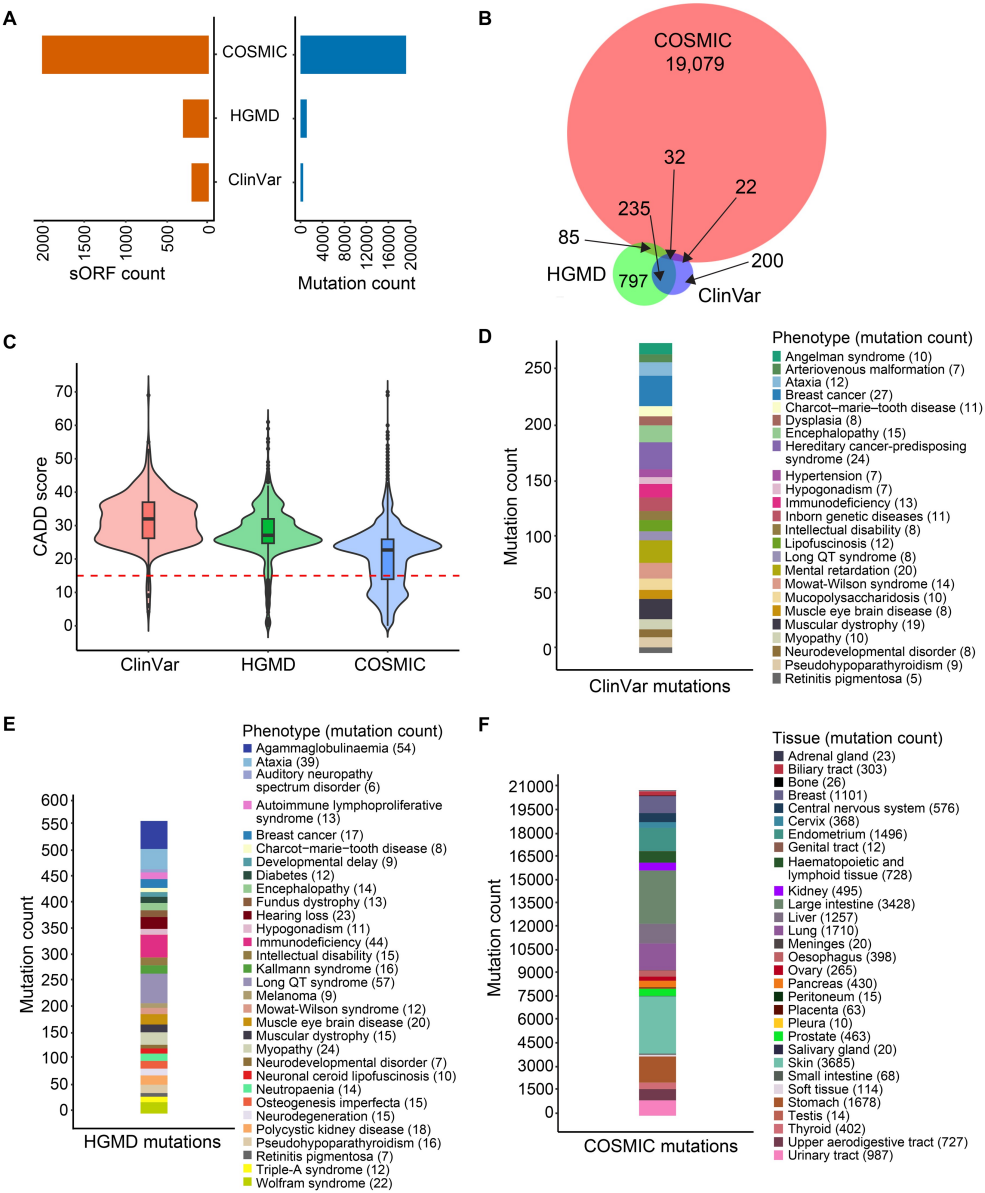
Disease-associated mutations in SEP-encoding genomic regions **A**. Number of sORFs overlapping pathogenic mutations from COSMIC, HGMD, and ClinVar. **B**. Venn diagram showing disease-associated mutations shared between COSMIC, ClinVar, and HGMD. **C**. Distribution of pathogenicity score (CADD) of sORFs overlapping mutations in ClinVar, COSMIC, and HGMD. **D**. Disease phenotypes associated with sORF mutations in ClinVar. **E**. Disease phenotypes associated with sORF mutations in HGMD. **F**. Number of sORF mutations associated with different cancer types based on COSMIC data. CADD, Combined Annotation Dependent Depletion; HGMD, Human Gene Mutation Database; COSMIC, Catalogue of Somatic Mutations in Cancer.

We mapped deleterious mutations from ClinVar, the Human Gene Mutation Database (HGMD), and the Catalogue of Somatic Mutations in Cancer (COSMIC) to sORF-containing regions in the genome. **Figure 5**A shows the number of mutations from ClinVar, HGMD, and COSMIC that map to sORF regions. A majority of these mutations were unique to individual databases (Figure 5B). Combined Annotation Dependent Depletion (CADD) mutation effect prediction analysis revealed that most mutations from ClinVar and HGMD were highly pathogenic while a subset of COSMIC mutations were below the pathogenicity threshold (Figure 5C). We observed that many of these mutations altered the protein sequences. The number of mutations in sORF regions associated with various disease phenotypes is shown in Figure 5D–F.

**Figure 6:**
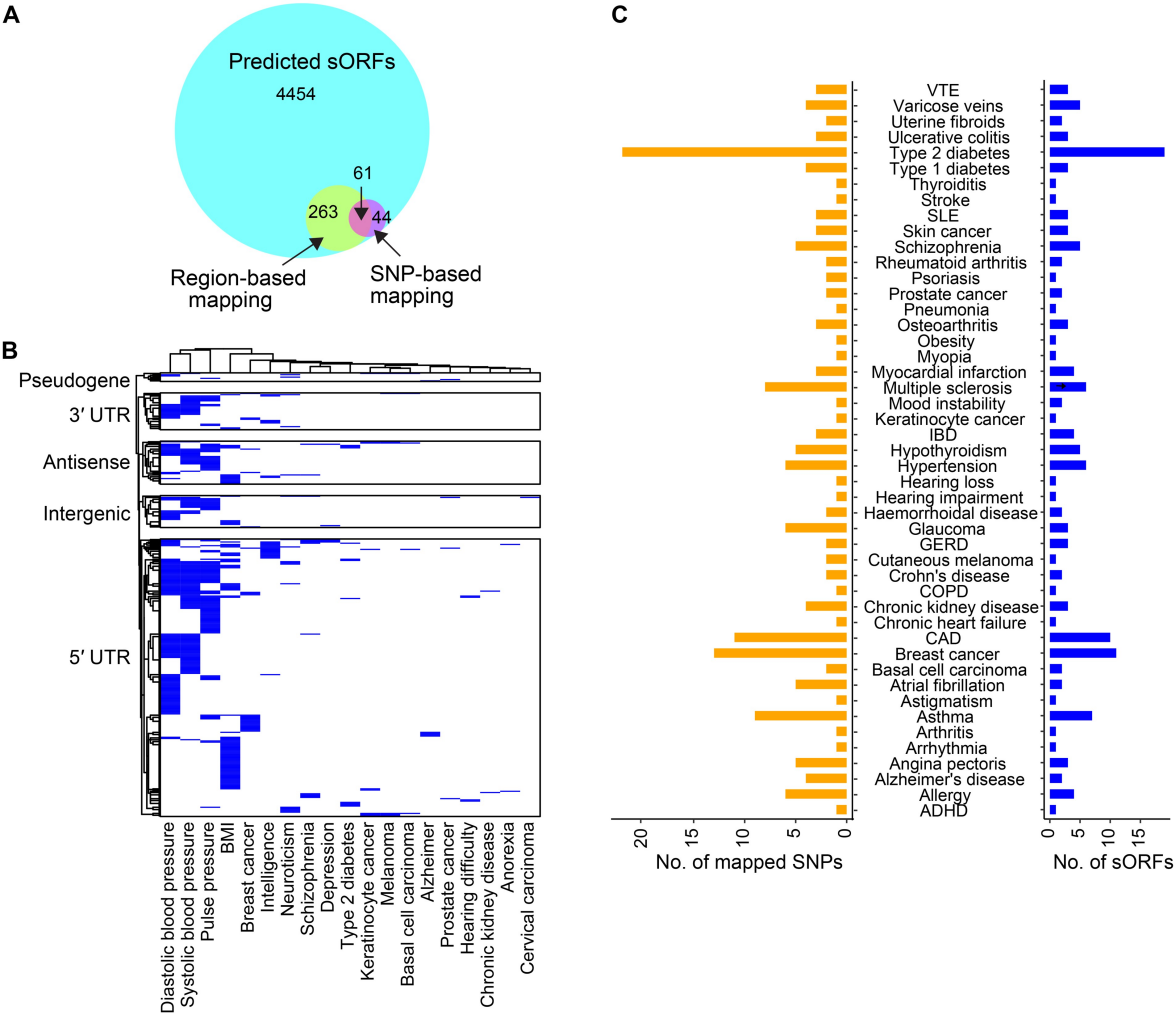
sORFs overlapping GWAS loci associated with different traits and disease phenotypes **A**. Proportion of sORFs identified to be associated with disease phenotypes using region-based and SNP-based mapping approaches. Outer circle represents the predicted sORFs and inner circles represent sORFs overlapping GWAS loci identified based on region-based and SNP-based mapping approaches. **B**. sORFs from regions with GWAS loci significantly associated with different traits and their associated biotypes. These comprised 30 3′ UTR, 226 5′ UTR, 35 antisense lncRNA, 26 intergenic lncRNA, and 7 pseudogene sORFs. **C**. Number of disease-associated SNPs mapped to sORF regions. SNP, single nucleotide polymorphism; BMI, body mass index; VTE, venous thromboembolism; SLE, systemic lupus erythematosus; IBD, inflammatory bowel disease; GERD, gastroesophageal reflux disease; COPD, chronic obstructive pulmonary disease; CAD, coronary artery disease; ADHD, attention deficit hyperactivity disorder.

Most disease-associated variants from genome-wide association studies (GWAS) are in non-coding regions of the human genome [38]. We carried out a region-based test using fastBAT to determine sORFs overlapping disease-associated GWAS loci. We identified 324 sORFs that were in disease-associated GWAS loci (**Figure 6**A), dominated by sORFs from UTRs (Figure 6B). Among these, 26 sORFs from intergenic lncRNAs were significantly associated with various traits including body mass index (BMI), systolic or diastolic blood pressure, and breast cancer. In addition, we employed a single nucleotide polymorphism (SNP)-based mapping approach and identified 105 sORFs associated with 47 traits. These SNPs were primarily linked to asthma, type 2 diabetes, breast cancer, coronary artery disease (CAD), multiple sclerosis, hypertension, and schizophrenia (Figure 6C). A list of all sORF candidates and their overlapping SNPs is provided in Table S7. These results are preliminary and require systematic investigation to determine their potential roles in the disease context.

### Function of lncRNA-encoded novel proteins

The role of several lncRNAs has been characterized through either overexpression systems or clustered regularly interspaced short palindromic repeat (CRISPR)-based knockout studies. Transcriptome profiling studies have also identified lncRNAs exhibiting differential expression patterns in various diseases, including cancers. We assessed whether our list of novel protein-coding sORFs included sORFs derived from lncRNAs with known function. We found protein-coding evidence for well-characterized lncRNAs including *GAS5*, *CASC15*, and *MALAT1* (Table S8). We identified protein-coding evidence for an sORF in the *NUTM2A-AS1* lncRNA. Interestingly, this sORF was one of the 57 ORFs that showed phenotypes in the CRISPR screening conducted by Prensner and his colleagues [39]. Systematic studies are required to evaluate whether these lncRNAs exert their functions through their translated products.

### SEPs are a source of MHC-I-presented peptides

**Figure 7:**
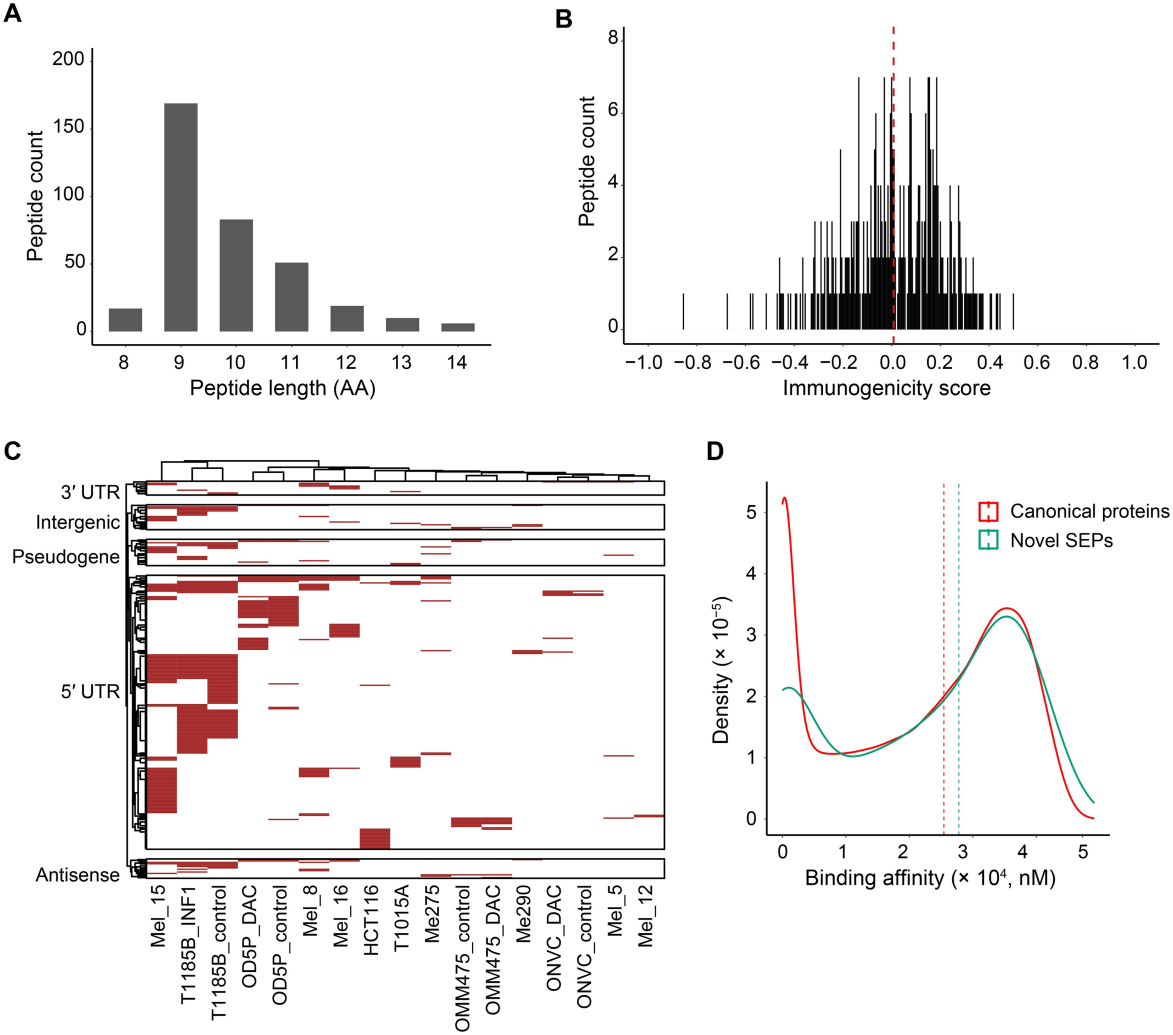
MHC-I-presented peptides derived from SEPs **A**. Length distribution of SEP-derived peptides presented by MHC-I. **B**. Immunogenicity score distribution of MHC-I-presented peptides derived from SEPs. **C**. MHC-I-presented peptides derived from SEPs and their associated biotypes. Each column represents data from a cancer cell line. This heatmap included peptides derived from 259 SEPs including 10 3′ UTR, 198 5′ UTR, 14 antisense lncRNA, 18 intergenic lncRNA, and 19 pseudogene SEPs. **D**. Binding affinity distribution of MHC-I-presented peptides derived from novel SEPs and canonical proteins. MHC-I, major histocompatibility complex class I.

Several studies in recent years have reported that peptides from noncanonical ORFs are presented by major histocompatibility complex class I (MHC-I) on the cell surface, expanding the repertoire of potential targetable epitopes for cancer immunotherapy [20]. We analyzed publicly available immunopeptidome datasets to determine whether any SEPs from our study were presented by MHC-I. We detected 355 MHC-I-presented peptides derived from 259 SEPs (Table S9). As expected, most of these peptides were 9-mers [40,41], though we also observed a subset of 8-, 10-, and 11-mers (**Figure 7**A). Immunogenicity analysis of these peptides revealed a varying degree of immunogenic potential (Figure 7B), with most peptides derived from SEPs encoded by sORFs from UTRs (Figure 7C). We next compared the MHC-I binding affinity of these peptides with those derived from canonical proteins. We expected genuine SEP-derived peptides to show comparable MHC-I binding affinity to those derived from canonical proteins. Interestingly, we observed similar binding affinity distribution between peptides derived from SEPs and canonical proteins (Figure 7D).

## Discussion

The human protein-coding gene catalog plays a central role in driving biomedical research. Almost all researchers use this catalog as a reference to study the molecular basis of health and disease. Current reagents including exon capture kits, antibodies, polymerase chain reaction (PCR) primers, small interfering RNAs (siRNAs), and guide RNAs are predominantly developed based on this reference catalog. Therefore, identification and annotation of all protein-coding genes is essential. In this study, we compiled putative sORFs located in ncRNAs and UTRs that are not annotated in reference databases, and established protein-coding evidence for these sORFs using publicly available Ribo-seq and proteomic datasets.

One of the important insights that we gained from compiling ribosome occupancy signals and protein expression patterns across multiple datasets is the stochastic nature of protein translation activity in most sORFs. Most ribosome occupancy signals in sORFs exhibited inconsistent expression patterns and showed greater dispersion than those in canonical ORFs in mRNAs. Similar observation has been reported by Erady and his colleagues [42]. Most ribosome occupancy signals were observed in UTRs. It is unclear what fraction of these sORFs could be noise due to leaky scanning as a result of ribosome engagement of these mRNAs for translation of canonical proteins and what fraction of these sORFs encode proteins that are involved in regulating cellular functions or have physiological roles.

Transcripts encoded by pseudogene regions were also a major source of sORFs. Pseudogenes are often considered non-functional sequences derived from canonical genes as a result of segmental duplication or reverse transcription of mRNAs and genomic integration. However, transcriptome sequencing studies have revealed extensive transcription of pseudogenes across different tissues. They also demonstrate tissue-specific expression patterns much like mRNAs. Previous studies have reported protein-coding potential of pseudogenes [43–45]. In our study, we identified SEPs encoded by 299 sORFs in transcripts from pseudogene regions.

While we could establish experimental evidence for the expression of these SEPs, their functions remain unknown. Most of these SEPs lack intact domains which makes it difficult to infer their potential functions based on computational analysis. We took advantage of GWAS locus catalog that lists genomic regions that are significantly associated with various traits and disease phenotypes. SEPs encoded in these regions can be investigated for their potential roles in regulating these phenotypes. We also found that peptides derived from many SEPs were presented by MHC-I. Previous studies have shown that peptides derived from noncanonical ORFs can be tumor-specific and can modulate immune response [20]. SEPs that show tumor-specific expression patterns can serve as a rich source of neoantigens for the development of immunotherapy strategies.

In the past decade, several SEPs have been functionally characterized using either loss-of-function (knockout/knockdown) or gain-of-function (overexpression/activation) studies [46]. It is also possible to gain better understanding of their disease-related roles based on differential expression patterns. For example, Cao et al. evaluated the expression patterns of 16 novel SEPs identified in their study and found 4 novel SEPs which were differentially expressed in leukemia [46,47]. Most SEPs form protein complexes with canonical proteins [48–50]. Therefore, identifying potential interacting partners may provide important insights into their roles. Functions and pathways regulated by their binding partners can help us assign functions to novel SEPs. For instance, co-immunoprecipitation (Co-IP) of the protein encoded by *MRI-2* identified two interacting proteins Ku70 and Ku80. These proteins are involved in non-homologous end joining (NHEJ) pathway, suggesting a potential role of MRI-2 in DNA repair pathway. This was further confirmed by Slavoff and his colleagues [13]. Another approach could be to tag proteins to study their subcellular localization and interactions. For example, Anderson et al. found that lncRNA-encoded myoregulin (MLN) expressed in *C2C12* myoblasts was enriched in subcellular fractions comprising sarcoplasmic reticulum membrane proteins. Co-IP experiments revealed that this protein forms a complex with sarcoplasmic/endoplasmic reticulum calcium ATPase: SERCA1, SERCA2a, and SERCA2b. MLN shares structural and functional similarities with phospholamban (PLN) and sarcolipin (SLN) which inhibit the Ca^2+^ uptake [51]. Recently, a high-throughput approach was used to functionally characterize sORFs [5,39]. Prensner et al. employed genome-wide CRISPR knockout of 553 noncanonical ORFs to investigate if they produce any phenotypic defects in human cancer cells, and they established protein-coding evidence for 257 candidates. Among these, 57 candidates were associated with viability defects [39].

There are several challenges associated with the identification and functional characterization of SEPs. Ribo-seq assays require rapid inhibition of ribosomes to capture particular disease/physiological states, which may lead to some inherent biases. One must be aware of the fact that not all ribosomal engagements are indicators of protein translation, nor will all ribosome-associated transcripts yield intact proteins [46]. Some studies have argued that ribosome occupancy alone is not a good indicator of protein-coding potential of a transcript [34]. SEPs are low-molecular-weight and low-abundance proteins, making their reproducible detection in proteomic assays challenging. Unlike canonical proteins, SEPs are highly unstable, which makes their functional validation difficult. Due to their small size, antibody-based validation becomes challenging. As many of them harbor transmembrane domains or form complexes with larger proteins, antibody-binding sites may be unavailable for immunoprecipitation [52]. Eliminating false positives from the hundreds of thousands of predicted noncanonical ORFs is a major challenge in small protein research. Our curated candidate set can help eliminate these false positives and serve as a resource for both proteogenomic studies and functional screens to decipher the roles of SEPs in cellular processes and human diseases.

In this study, we utilized publicly available datasets that were generated from different tissues/cell types using very different methods. Differences in sample processing protocols themselves may introduce huge variations that affect dataset quality. Transcriptomic, Ribo-seq, and proteomic data generated from the same samples would be ideal for this type of analysis. Furthermore, it is desirable to have such datasets generated from multiple biological replicates for each tissue/cell type. Currently, most available Ribo-seq datasets are derived from cell lines. Moreover, these datasets are limited by the lack of large-scale profiling across diverse human tissues, making them unsuitable for identifying sORFs with tissue-restricted expression patterns. Additionally, the cutoff used in our study to eliminate false-positive candidates may exclude some genuine hits. We restricted our analysis to ORFs that have at least 90 codons with an AUG initiation codon, which may potentially exclude legitimate sORFs that are shorter than 90 codons and originate from noncanonical start sites.

## Materials and methods

### Merged transcriptome database

We constructed a comprehensive, non-redundant catalog of transcripts by merging RNA sequences from GENCODE (v33), LNCipedia (v5.2), and NONCODE (v5). Read-through transcripts were removed, resulting in a final set of 384,193 transcripts (Table S2).

### Predicted SEP database

All transcripts were conceptually translated in three reading frames. Protein sequences derived from ORFs ≥ 30 codons were used to build the predicted SEP database.

### Sequence conservation analysis within the sORF regions

We utilized genome-wide conservation data from the University of California Santa Cruz (UCSC) Genome Browser [33], which provides phyloP-based conservation scores for each nucleotide across 100 vertebrate species via multiple sequence alignment. Positions with positive scores were conserved. First, we calculated the average conservation score for nucleotides within the CDS regions of canonical genes and determined their median value (1.42) to establish the conservation score cutoff. Next, we evaluated the proportion of conserved nucleotides (positions with positive scores) in the CDS regions of canonical genes across vertebrates, and found that 75% of the canonical genes contained ≥ 73% conserved nucleotides within the CDS regions while the remaining 25% showed < 73% conserved nucleotides. Using these two parameters, we classified novel sORFs as those exhibiting both ≥ 73% conserved nucleotides and an average conservation score of 1.42.

### Post-processing of predicted SEPs

SEPs with ≥ 90% sequence identity were clustered using Cluster Database at High Identity with Tolerance (CD-HIT) [27,53], and the longest SEP per cluster was retained.

### Ribo-seq data processing and analysis

Raw FASTQ files of 45 publicly available Ribo-seq datasets comprising 637 samples were downloaded from the Sequence Read Archive (SRA) (Table S4). Initial quality control (QC) was performed using FastQC (v0.11.8) and MultiQC (v1.7). Reads with adapter sequences were trimmed using Cutadapt (v1.16) followed by the depletion of ribosomal RNA (rRNA) sequences using Bowtie2 (v2.2.9). Sequences that remained after rRNA depletion were aligned to the human reference genome (GRCh38) in transcriptome-guided mode using STAR (v2.7.1a) with two-pass alignment, retaining only uniquely mapped reads. The resulting Binary Alignment Map (BAM) files were provided as input to Ribotricer (v1.3.2) [54]. Typical ribosome footprints range from 29 to 30 nt. However, their lengths may be affected by library preparation protocols. Therefore, we considered the RPFs with lengths of 25–34 nt [55]. Ribotricer mapped reads from RPFs against a precompiled database of ORFs. Predicted ORFs and ORFs that encode canonical proteins were provided as a reference, and Ribotricer was run with default settings using a *Homo sapiens*-specific phase score cutoff of 0.440. Raw RPF read count was converted into TPM. Unlike RNA sequencing (RNA-seq), we used ORF length instead of transcript length.

### Dispersion analysis of predicted sORFs with ribosome occupancy signals

Dispersion in ribosome footprints in predicted sORFs compared to ORFs in canonical protein-coding genes was assessed by comparing RPF-derived read counts across samples. Ribosome profiling datasets generated by van Heesch et al. [9] and Battle et al. [56] were used for the analysis.Transcripts with maximum RPF read counts were considered in cases where a gene had multiple isoforms. NB models were used to model RPF read counts using the total number of reads (log) as an offset for each gene to estimate the mean rate (read counts in ORF divided by total reads in sample) and dispersion parameter alpha (α). NB2 parametrization with mean μ and variance $\mu +\alpha \mu^{2}=\mu (1+\alpha \mu )\;$where α>0 was used. Estimated dispersion parameters and means were visualized and compared between predicted sORFs and canonical protein-coding ORFs. R (v4.1.0) and the R statistical package glmmTMB (v1.1.4) were used for analysis. A higher α indicates greater excess variation relative to the Poisson distribution in expression across samples.

### Proteomic analysis

Proteomic datasets from healthy and cancerous tissues were downloaded from public repositories (Table S5). The data were searched using the SEQUEST HT search algorithm through Proteome Discoverer (Thermo Fisher Scientific, Bremen, Germany). A custom protein database was generated by combining protein sequences from candidate sORFs and canonical protein sequences from UniProtKB. Carbamidomethylation of cysteine was specified as a fixed modification, while oxidation of methionine and acetylation of protein N-termini were set as variable modifications. The precursor ion mass tolerance was set to 10 ppm and 20 ppm for Label-Free Quantification (LFQ) and Tandem Mass Tag (TMT) data, respectively. Fragment ion mass tolerance was set to 0.05 Da. A 1% false discovery rate (FDR) was set at both protein and peptide levels. Peptides identified with XCorr ≥ 2 were considered for downstream analysis. Uniquely mapped sORF-derived peptides ranging from 7 to 20 amino acids (AA) were subjected to Basic Local Alignment Search Tool for Proteins (BLASTP) analysis with default parameters, including an option that automatically adjusts the parameters for short sequences. Peptides with less than two mismatches to any known proteins in the human RefSeq protein database were filtered out as they could potentially result from SNPs, while peptides with two or more mismatches were retained as two mismatches in a short stretch of 10–20 residues are unlikely to be due to SNPs. SEPs with peptides meeting the criterion were considered novel proteins. SEPs that were detected in at least three samples with ≥ 10 PSMs were categorized as high-confidence candidates.

### Differential expression analysis of identified SEPs

The relative abundance of proteins and novel SEPs in cancers was determined based on TMT reporter ion intensity ratios. Differentially expressed proteins in tumor samples compared to normal samples were identified for four cancer datasets in CPTAC including HNSCC, LUAD, LSCC, and liver cancer. Proteins absent in > 50% of samples within a dataset were excluded. Raw intensity values were normalized using the probabilistic quotient normalization (PQN) method to correct for sample concentration variation, followed by log_2_ transformation [57]. Missing values were imputed using a Gibbs sampler-based left-censored approach [58]. Linear mixed effects models (implemented in the R package *lme4*) were used to compare intensities between normal and tumor samples (paired and unpaired), accounting for within-individual correlations in paired samples. *P* values were adjusted using the Benjamini–Hochberg method [59].

### RNA-seq analysis

Precomputed TPM values from the Genotype-Tissue Expression (GTEx) Project (v8_RSEMv1.3.0) were utilized. GTEx employs GENCODE v26 reference annotations. TPM values for transcript IDs matching with GENCODE v33 (the reference used in our study) were retrieved for transcriptomic analysis. Transcript biotype annotations were based on GENCODE (v33).

### Sequence feature analysis of SEPs

Protein localization was predicted using TargetP-2.0 by specifying the organism group as non-plant [60]. Prediction of signal peptides and cleavage sites was performed using SignalP 6.0 [61]. In addition, TMHMM 2.0 and DeepTMHMM were used to identify transmembrane helices in SEPs [62].

### GWAS analysis

We employed two approaches to identify sORFs in GWAS loci significantly associated with specific traits or disease phenotypes: a region-based approach and a SNP-based mapping approach. For the region-based approach, we utilized the fastBAT method [63] to identify disease-associated sORF regions, which computes aggregated effects of a set of SNPs within or near genes by calculating association *P* values using GWAS summary-level data and incorporating linkage disequilibrium (LD) correlations between SNPs from reference samples with individual-level genotypes (using UK Biobank LD estimates as the reference panel). We analyzed individual summary statistics files from 27 high-profile studies, with predicted sORF regions as targets. The details of the traits considered in the analysis are provided in Table S7. Association *P* values for each set of SNPs were corrected for multiple comparisons using the Bonferroni method by dividing each *P* value by the number of tests conducted across all traits. Regions exhibiting *P* ≤ 0.7 × 10^−8^ were considered significant associations. For the SNP-based mapping approach, we used significantly associated SNPs identified in various GWAS studies that are reported in the GWAS Catalog (v1.0.2). Studies with a sample size of at least 10,000 were considered for analysis (Table S7). SNPs mapping to the candidate regions with *P* ≤ 5 × 10^−8^ were considered significantly associated.

### Mutation analysis

Disease-associated mutations from ClinVar (release date: May 2, 2022) and the HGMD database were mapped using BEDTools. Single nucleotide variants (SNVs) with pathogenicity or clinical significance were retained. A similar analysis was performed for somatic SNVs from the COSMIC (v95) database of non-coding variants, which provides a functional score (ranging from 0 to 1) for individual variants calculated using the FATHMM-MKL algorithm [64]. SNVs with scores > 0.7 were considered functionally significant. We considered all somatic SNVs meeting the above qualification criteria. Furthermore, the pathogenicity of mapped SNVs was assessed using the CADD webserver (GRCh38-v1.6) [65,66], which provides normalized Phred-scaled scores (C-scores) ranging from 1 to 99. Scores > 10 indicate SNVs among the top 10% most deleterious substitutions, whereas scores > 20 indicate SNVs among the top 1% most deleterious substitutions. As recommended by CADD, we considered SNVs with scores > 15 as deleterious.

### Immunopeptidomic analysis

We utilized publicly accessible MS-based immunopeptidomic datasets to investigate whether sORF-derived peptides are presented by MHC-I (Table S9). These datasets were generated through immunoaffinity capture of human leukocyte antigen (HLA)*-*bound peptides followed by liquid chromatography (LC)-MS/MS analysis.

Proteomic searches were performed using PEAKS. Immunopeptidomic datasets were searched against a custom database containing predicted SEPs, UniProt canonical proteins, and common contaminants. Oxidation of methionine, acetylation of protein N-termini, and carbamidomethylation were set as variable modifications. Parent mass error tolerance and fragment mass error tolerance were set to 20 ppm and 0.05 Da, respectively. No enzyme was specified. Unique peptides (8–15 AA in length) detected at a 1% FDR threshold were considered for further analysis [20]. In second-pass analyses, identified peptides were searched using BLASTP against the non-redundant protein database to eliminate those sharing exact similarity with known proteins. The binding affinity of uniquely mapped HLA peptides was determined using NetMHCpan (v4.1) by providing HLA allele information for corresponding cell types (Table S9) [67]. The immunogenicity scores of these peptides were calculated using the Immune Epitope Database (IEDB) web server [68], which predicts the ability of HLA peptides to elicit an immune response based on their amino acid composition. Peptides with higher scores are more likely to be immunogenic.

### Data visualization

All plots were generated using the ggplot2 R package [69]. Heatmaps were created using the ComplexHeatmap R package [70]. Venn diagrams were generated using the web-based BioVenn application [71].

## Supplementary Material

qzaf004_Supplementary_Data

## CRediT author statement

**Hitesh Kore:** Conceptualization, Investigation, Methodology, Data curation, Writing – original draft. **Satomi Okano:** Methodology, Formal analysis, Visualization. **Keshava K. Datta:** Conceptualization, Writing – review & editing. **Jackson Thorp:** Formal analysis. **Parthiban Periasamy:** Formal analysis. **Mayur Divate:** Visualization. **Upekha Liyanage:** Data curation, Resources. **Gunter Hartel:** Supervision, Formal analysis. **Shivashankar H Nagaraj:** Resources, Writing – review & editing, Supervision. **Harsha Gowda:** Conceptualization, Methodology, Supervision, Project administration, Funding acquisition, Resources, Writing – review & editing. All authors have read and approved the final manuscript.
