# Supplementary material for: Identification of Small Open Reading Frame-encoded Proteins in the Human Genome
Source: Genomics Proteomics Bioinformatics. 2025 Feb 7;23(1):qzaf004. doi: 10.1093/gpbjnl/qzaf004 (PMC12236067; doi:10.1093/gpbjnl/qzaf004)
Supplement: qzaf004_Supplementary_Data [file qzaf004_supplementary_data.zip › Supplementary material captions.docx]

**Supplementary material**

**File S1 Supplementary data and methods**

**Figure S1 Distinguishing features of ORFs in mRNAs and lncRNAs**

**A.** Number of exons per transcript in mRNAs and lncRNAs. **B.** Length of longest ORF in mRNAs and lncRNAs. **C.** Average conservation score of predicted sORFs in lncRNAs and canonical ORFs in mRNAs. **D.** Proportion of nucleotides in predicted sORFs and canonical ORFs that show conservation across vertebrates.

**Figure S2 Conservation in CDS region of canonical protein-coding genes and ORFs in transcripts that were previously designated as lncRNAs**

**A.** Representative examples of conserved (*CHAC2*) and poorly conserved (*SPANXC*, *ULBP3*, and *RHOXF2B*) genes in the human genome. **B.** Representative examples of genes that were previously designated as lncRNA genes but were later found to be protein-coding.

**Figure S3 ORFs with ribosome occupancy signal**

**A.** Number of samples with ribosome occupancy signal in canonical ORFs and predicted sORFs. **B.** Number of datasets with ribosome occupancy signal in canonical ORFs and predicted sORFs.

**Figure S4 RPF abundance in high and low confidence sORF candidates compared to canonical ORFs in mRNAs**

**A**. RPF abundance in sORFs and canonical ORFs in mRNAs based on Ribo-seq data from Heesch and his colleagues. Data represented in each square corresponds to an individual sample. Statistical significance was calculated using Wilcoxon test. **B.** RPF abundance in sORFs and canonical ORFs in mRNAs based on Ribo-seq data from Costa and his colleagues. Statistical significance was calculated using Wilcoxon test. **C.** RPF abundance in sORFs and canonical ORF in mRNAs based on Ribo-seq data from Loayza-Puch and his colleagues. Statistical significance was calculated using Wilcoxon test. Ribo-seq, ribosome profiling.

**Figure S5 Representative examples of sORFs supported by Ribo-seq evidence**

**Figure S6 MS/MS mirror plots showing predicted and observed fragmentation pattern of peptides from SEPs**

MS/MS, tandem mass spectrometry.

**Figure S7 Expression abundance of mRNAs and novel sORF containing transcripts across human tissues in GTEx datasets**

GTEx, Genotype-Tissue Expression.

**Figure S8 SEPs with domain signatures and their associated biotypes**

**A.** Number of SEPs with domain signatures and their associated biotypes. **B.** Representative examples of SEPs with conserved domains.

**Table S1 List of mRNAs that were previously annotated as lncRNAs in Ensembl**

**Table S2 Transcripts in merged transcriptome database**

**Table S3 Number of candidate ORFs and their biotypes**

**Table S4 Ribo-seq datasets used and RPF abundance in sORFs**

**Table S5 Proteomics datasets used and identified peptides from SEPs**

**Table S6**  **List of novel sORFs with Ribo-seq or proteomics evidence**

**Table S7 List of GWAS studies and GWAS loci mapped to sORF regions**

**Table S8 Examples of lncRNAs with known function for which we identified protein-coding evidence**

**Table S9 SEP derived peptides presented by MHC-I complex**
