# Supplementary material for: Identification of Small Open Reading Frame-encoded Proteins in the Human Genome
Source: Genomics Proteomics Bioinformatics. 2025 Feb 7;23(1):qzaf004. doi: 10.1093/gpbjnl/qzaf004 (PMC12236067; doi:10.1093/gpbjnl/qzaf004)
Supplement: qzaf004_Supplementary_Data [file qzaf004_supplementary_data.zip › File S1.docx]

**File S1 Supplementary data and methods**

**Ribosome profiling analysis**

We considered open reading frames (ORFs) that qualify phase score cut off of 0.440. Ribotricer considers phase score which accounts for 3 nucleotide periodicity by projecting 3-dimensional (3D) read count vectors into 2-dimensional (2D) unit vectors. High phase score indicates strong consistency in periodicity, *i.e.*, high–low–low pattern for most codons in ORF. As can be seen from Figure SS1A, canonical ORFs have higher phase score distribution compared to predicted novel ORFs, whereas novel ORFs showed better codon coverage (which represents number of p sites covered by each ORF) and read density compared to canonical ORFs (Figure SS1B and C). This could be because of their short ORF length, suggesting that read density and codon coverage alone are not good indicators to determine coding potential of ORFs based on ribosome occupancy. Phase score corrects for higher abundance due to unknown artifacts or noise due to ribosome decoding speed, ribosomal pause, or presence of ribosomal fragments. Therefore, we decided not to filter the predicted ORFs based on minimum reads in individual samples. Instead, we eliminated ORFs that are detected in fewer number of samples and insufficient ribosome-protected fragment (RPF) abundance [median RPF abundance ≤ 1 transcripts per million (TPM) in expressed samples].

**
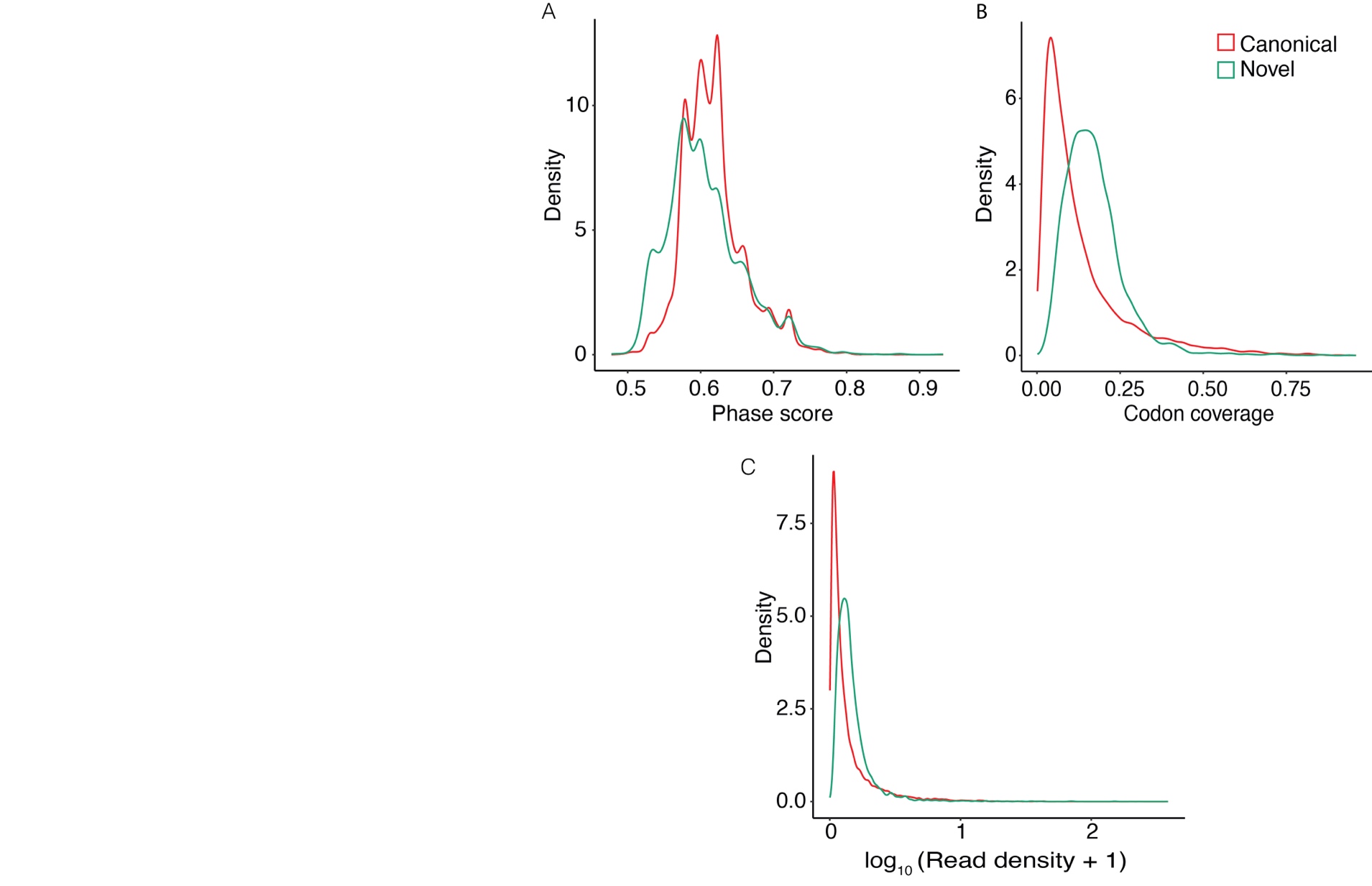
**

**Figure SS1 Comparison of phase score, codon coverage and read density in predicted and canonical ORFs.**

**A.** Distribution of phase score in predicted ORFs compared to canonical ORFs. **B.** Distribution of codon coverage in predicted ORFs compared to canonical ORFs. **C.** Distribution of read density in predicted ORFs compared to canonical ORFs.

We derived cut off for ribosome profiling (Ribo-seq) analysis based on the distribution of canonical ORFs across samples and datasets. Using the criterion of ribosome occupancy in at least 40 samples and 4 independent studies, we could detect 83% of total detected canonical ORFs. This was an optimum cut off that minimized inclusion of false positives thereby maximizing the detection of genuine candidates.

**
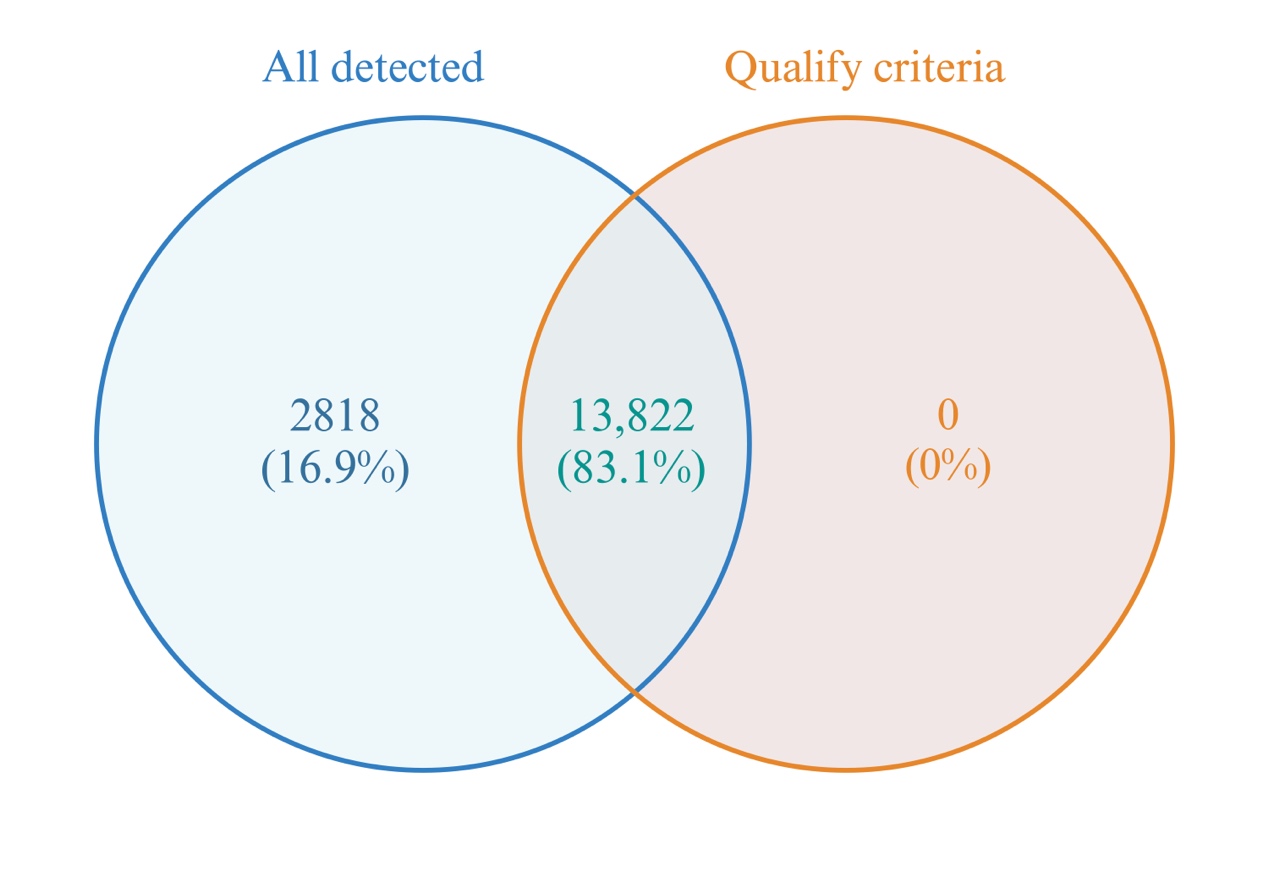
**

**Figure SS2 Proportion of canonical ORFs qualifying minimum ribosome occupancy criteria across samples and datasets**

Number of canonical ORFs detected in each sample are shown in figure below.


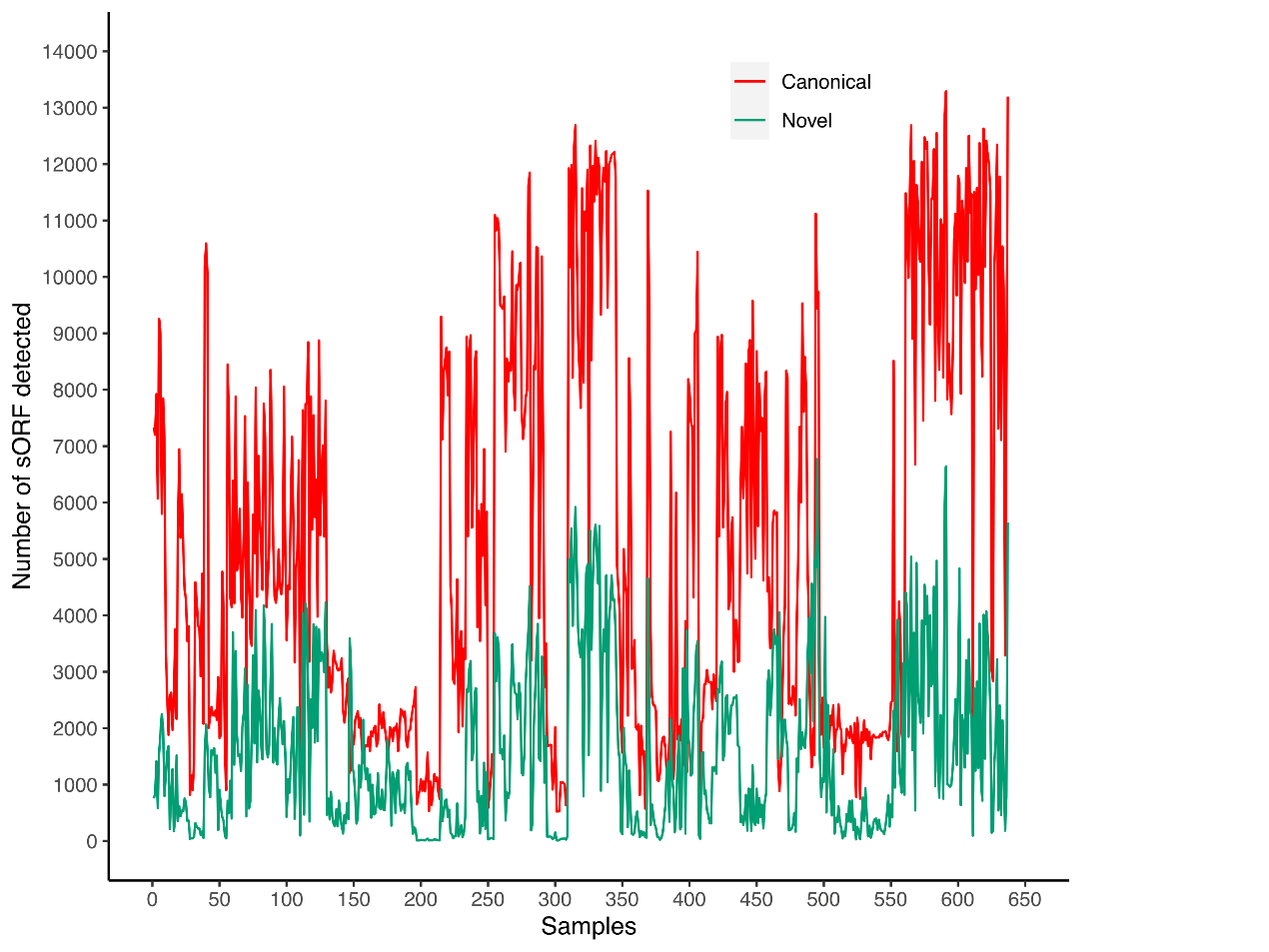


**Figure SS3 Distribution of detected canonical and novel ORFs across samples in Ribo-seq datasets**

sORFs, small open reading frames.

**BLAST parameters used to identify uniquely mapped peptides**

**
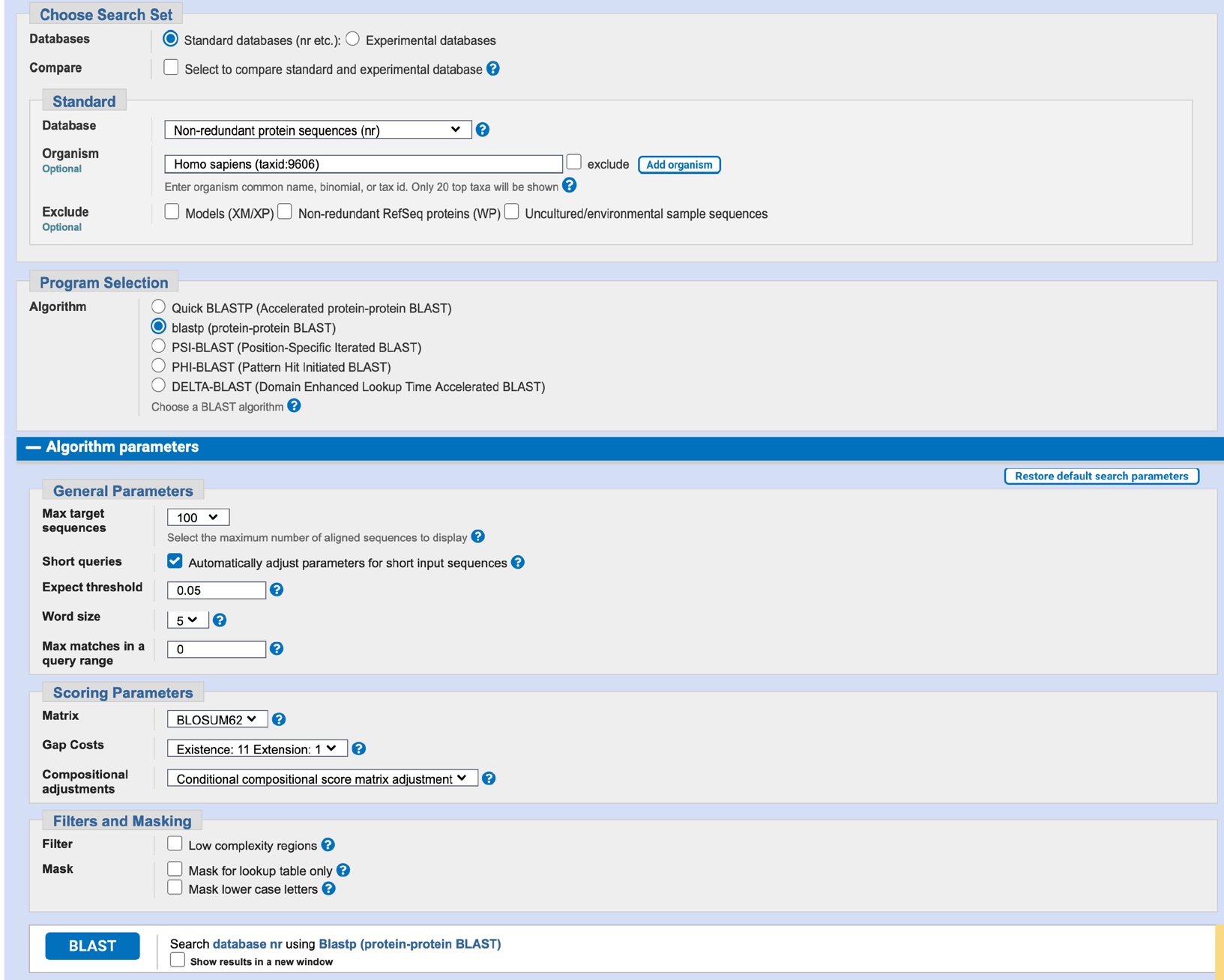
**Basic Local Alignment Search Tool (BLAST) was run using default parameters with an option that allows BLAST to adjust parameters for short sequences. The screenshot of BLAST with parameter settings is provided below.

**Figure SS4 Parameters used for BLAST analysis**

**Distribution of detected canonical proteins across chromosomes**


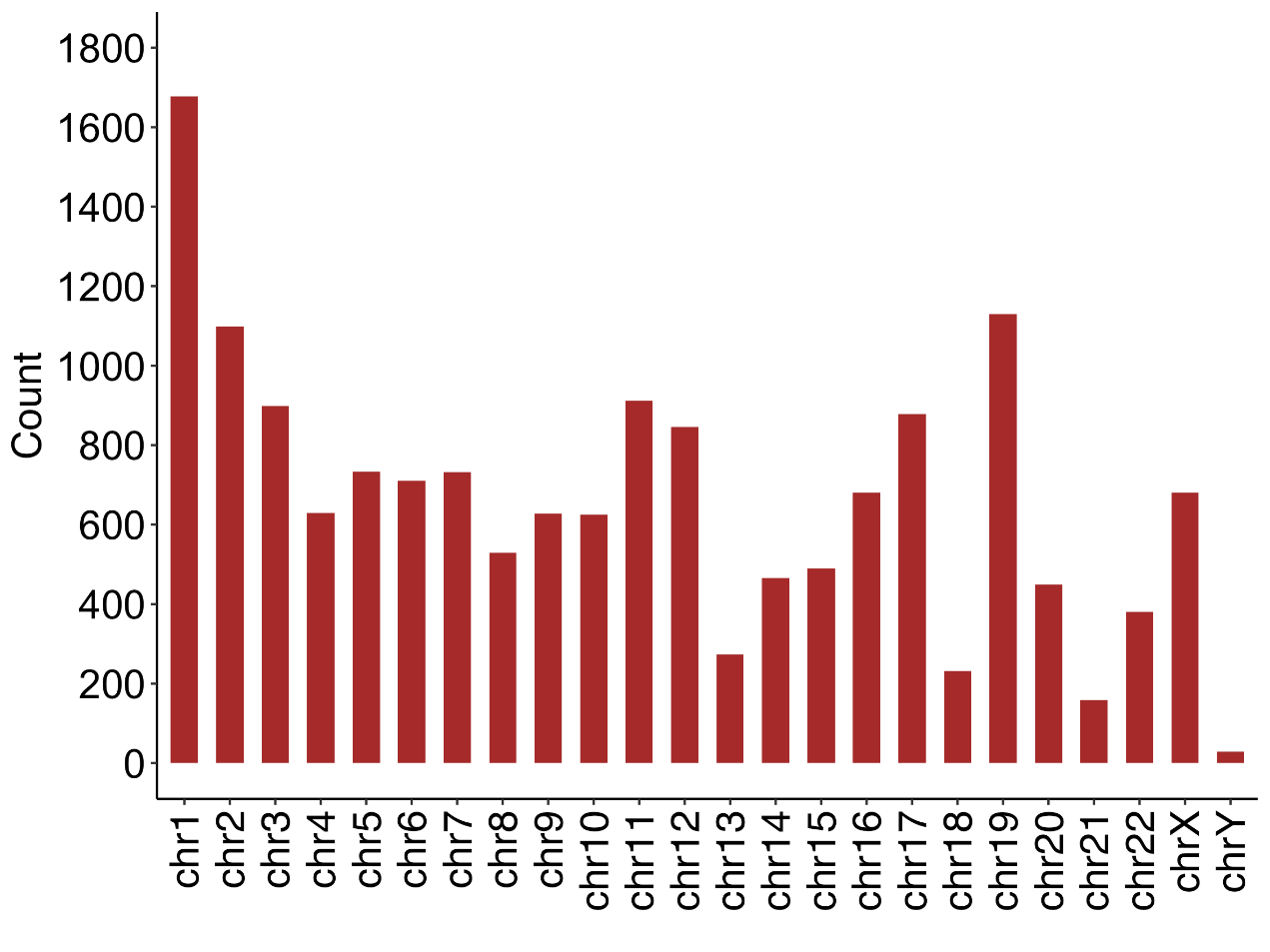


**Figure SS5 Number of canonical proteins encoded by different chromosomes**

This includes a total of 16,750 proteins comprising 12,941 commonly detected and 2939 with only proteomics and 870 with only Ribo-seq evidence. Proteins encoded by scaffolds were excluded. ORFs with Ribo-seq evidence include those that are detected in at least 40 samples and 4 independent studies. ORFs, open reading frames.

**Relationship between dispersion parameter (alpha) and negative binomial mean**

We observed negative correlation between dispersion and negative binomial (NB) mean (Figure SS6).

**
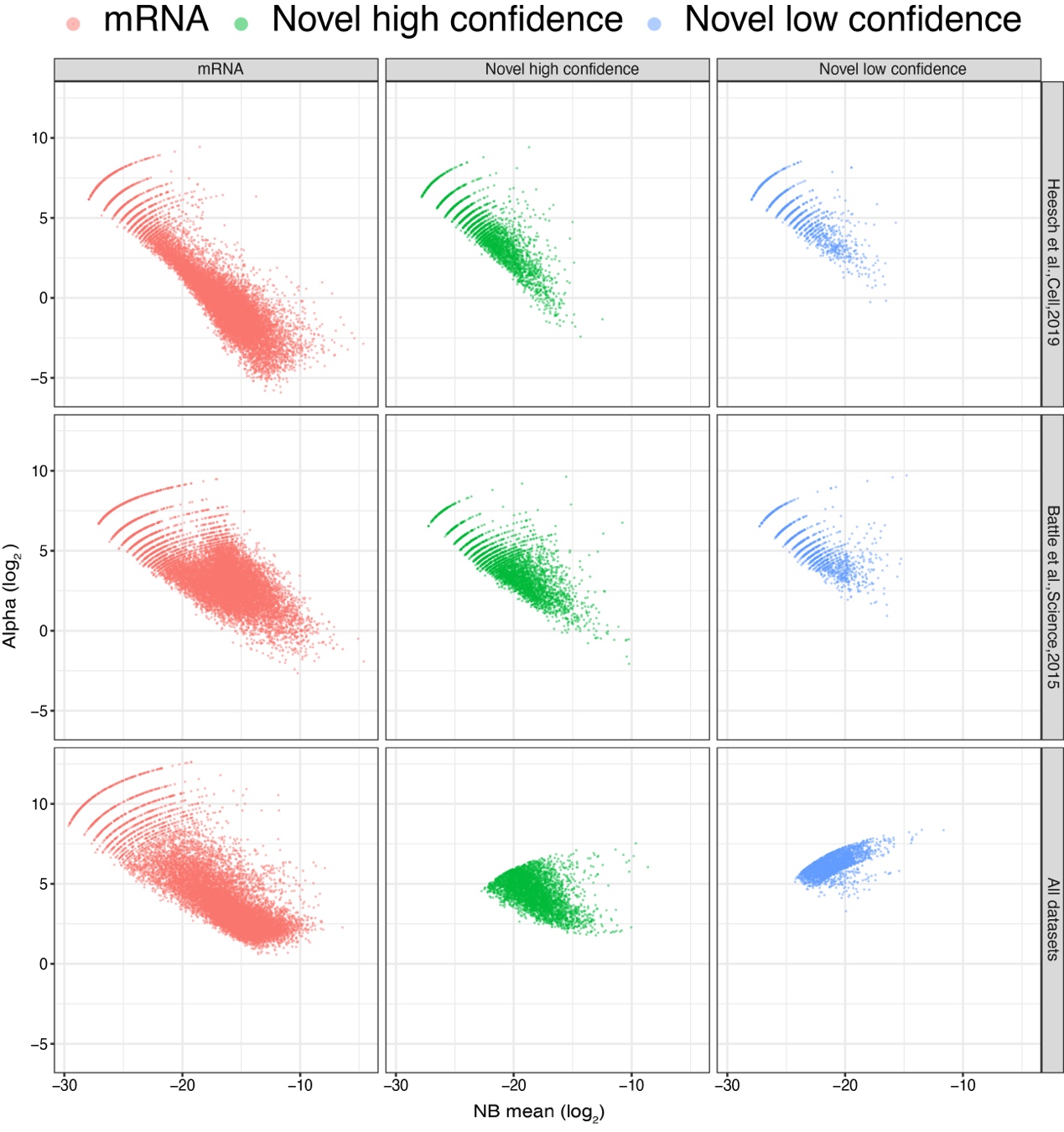
**

**Figure SS6 Relationship between dispersion parameter Alpha and NB mean**

Higher Alpha indicates higher dispersion. Higher NB mean indicates more RFP abundance. Each data point indicates ORF. mRNA, messenger RNA; RPF, ribosome-protected fragment; NB, negative binomial.

We further compared the NB mean and dispersion (in log scale) in coding sequence (CDS) regions of messenger RNA (mRNA) to predicted low and high confidence candidates using linear regression. *P* values are adjusted using Sidak method. Table SS1 shows geometric mean fold change (GMFC) with 95% confidence interval (CI).

**Table SS1 Comparison of negative binomial means and dispersion parameters (Alpha) between predicted low and high confidence candidates and CDS regions in mRNAs**

| **Parameter** | **Dataset** | **Comparison** | **GMFC (95% CI)** | **Sidak adj**  ***P* value** |
| --- | --- | --- | --- | --- |
| Mean | Heesch et al., cell, 2019 | Novel-low/mRNA CDS | 0.01 (95% CI: 0.01, 0.01) | < 0.0001 |
| Mean | Heesch et al., cell,2019 | Novel-high/mRNA CDS | 0.04 (95% CI: 0.03, 0.04) | <0.0001 |
| Mean | Heesch et al., cell, 2019 | Novel-high/Novel-low | 3.43 (95% CI: 2.84, 4.14) | < 0.0001 |
| Mean | Battle et al., Science, 2015 | Novel-low/mRNA CDS | 0.02 (95% CI: 0.02, 0.03) | < 0.0001 |
| Mean | Battle et al., Science, 2015 | Novel-high/mRNA CDS | 0.13 (95% CI: 0.11, 0.14) | < 0.0001 |
| Mean | Battle et al., Science, 2015 | Novel-high/Novel-low | 5.83 (95% CI: 4.91, 6.94) | < 0.0001 |
| Mean | All datasets | Novel-low/mRNA CDS | 0.05 (95% CI: 0.04, 0.05) | < 0.0001 |
| Mean | All datasets | Novel-high/mRNA CDS | 0.32 (95% CI: 0.3, 0.35) | < 0.0001 |
| Mean | All datasets | Novel-high/Novel-low | 6.66 (95% CI: 5.85, 7.59) | < 0.0001 |
| Alpha | Heesch et al., cell, 2019 | Novel-low/mRNA CDS | 27.13 (95% CI: 24.02, 30.63) | < 0.0001 |
| Alpha | Heesch et al., cell, 2019 | Novel-high/mRNA CDS | 11.43 (95% CI: 10.53, 12.41) | < 0.0001 |
| Alpha | Heesch et al., cell, 2019 | Novel-high/Novel-low | 0.42 (95% CI: 0.37, 0.48) | < 0.0001 |
| Alpha | Battle et al., Science, 2015 | Novel-low/mRNA CDS | 3.33 (95% CI: 3.08, 3.6) | < 0.0001 |
| Alpha | Battle et al., Science, 2015 | Novel-high/mRNA CDS | 1.38 (95% CI: 1.31, 1.45) | < 0.0001 |
| Alpha | Battle et al., Science, 2015 | Novel-high/Novel-low | 0.41 (95% CI: 0.38, 0.45) | < 0.0001 |
| Alpha | All datasets | Novel-low/mRNA CDS | 4.69 (95% CI: 4.39, 5.01) | < 0.0001 |
| Alpha | All datasets | Novel-high/mRNA CDS | 1.46 (95% CI: 1.38, 1.54) | < 0.0001 |
| Alpha | All datasets | Novel-high/ Novel-low | 0.31 (95% CI: 0.29, 0.34) | < 0.0001 |

*Note*: For instance, consider group A and group B where GMFC = 1, both the groups have same geometric mean. GMFC < 1 (group A < group B), GMFC closer to one means there is lesser difference in A and B. GMFC >1 (group A > group B), geometric mean A is higher than B. CI, confidence interval; CDS, coding sequence; GMFC, geometric mean fold change; mRNA, messenger RNA.

In case of NB mean, higher GMFC is observed in Novel-high/mRNA CDS (closer to 1) than Novel-low/mRNA CDS across all 3 comparisons, suggesting high confidence candidates following closer distribution of NB mean to CDS in mRNAs. There is higher difference observed in Novel-high /Novel-low (> 1), indicating high confidence candidates have higher GMFC.

In case of Alpha, higher GMFC is observed in Novel-low/mRNA CDS compared to Novel-high/mRNA CDS, indicating Novel-high showing lesser dispersion from mRNA CDS.

**Table SS2 Geometric means of estimated negative binomial means and dispersion parameter, Alpha in predicted low and high confidence candidates and CDS regions in mRNAs**

| **Dataset** | **Group** | **NB mean geometric mean (× 10^−6^) (95% CI)** | **Alpha geometric mean (95% CI)** |
| --- | --- | --- | --- |
| Heesch et al., cell,2019 | mRNA CDS | 6.51 (95% CI: 6.28, 6.78) | 1.25 (95% CI: 1.21, 1.28) |
| Heesch et al., cell,2019 | Novel-high | 0.25 (95% CI: 0.23, 0.28) | 14.32 (95% CI: 13.45, 15.24) |
| Heesch et al., cell,2019 | Novel-low | 0.07 (95% CI: 0.06, 0.08) | 33.82 (95% CI: 30.7, 37.27) |
| Battle et al., Science,2015 | mRNA CDS | 7.42 (95% CI: 7.12, 7.74) | 10.2 (95% CI: 9.99, 10.41) |
| Battle et al., Science,2015 | Novel-high | 0.95 (95% CI: 0.88, 1.02) | 14.03 (95% CI: 13.55, 14.62) |
| Battle et al., Science,2015 | Novel-low | 0.16 (95% CI: 0.14, 0.18) | 33.82 (95% CI: 31.78, 36) |
| All datasets | mRNA CDS | 9.86 (95% CI: 9.52, 10.14) | 15.45 (95% CI: 15.14, 15.78) |
| All datasets | Novel-high | 3.19 (95% CI: 2.99, 3.41) | 22.63 (95% CI: 21.71, 23.43) |
| All datasets | Novel-low | 0.48 (95% CI: 0.44, 0.52) | 72.5 (95% CI: 69.07, 76.11) |

**Differentially expressed sORFs encoded proteins (SEPs) across different cancer types**

Below table describes the details of tissues.

**Table SS3 Cancer datasets considered for differential expression analysis**

| **Cancer type** | **Study (PMID)** | **Normal** | **Tumor** | **Paired samples** | **Tissue site** |
| --- | --- | --- | --- | --- | --- |
| HNSCC | 33417831 | 67 | 108 | 64 | Oral cavity and larynx |
| LUAD | 32649874 | 101 | 109 | 100 | Lung |
| Liver cancer | 31585088 | 150 | 150 | 148 | Liver |
| LSCC | 34358469 | 102 | 110 | 102 | Bronchus and lung |

*Note*: Poor quality samples were excluded from the analysis. LUAD, lung adenocarcinoma; LSCC, lung squamous cell carcinoma; HNSCC, head and neck squamous cell carcinoma.


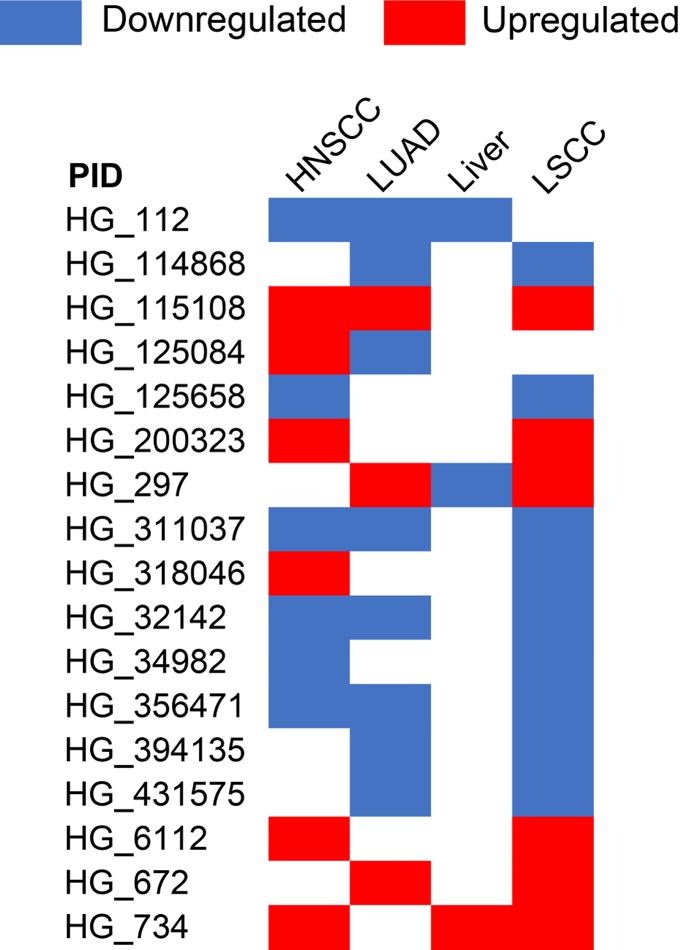


**Figure SS7 Differentially expressed SEPs across multiple cancer types**

SEPs, sORFs encoded proteins; LUAD, lung adenocarcinoma; LSCC, lung squamous cell carcinoma; HNSCC, head and neck squamous cell carcinoma.

**Overlap of candidates identified in Ribo-seq and proteomics analysis**

Out of 17,175 total detected candidates, we identified 266 candidates with both Ribo-seq and proteomics evidence.

Out of 4822 predicted high confidence candidates, 176 candidates were commonly detected ORFs in Ribo-seq and proteomics analysis.


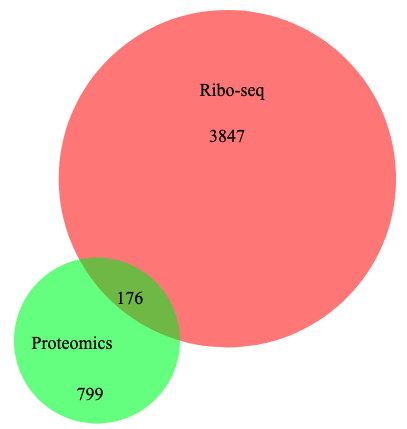


**Figure SS8 Overlap of ORFs detected with Ribo-seq and proteomic evidence**

High confidence candidates were considered in the overlap analysis.

**Overlap of predicted ORFs with catalog of noncanonical ORFs provided by Mudge and his colleagues**

**
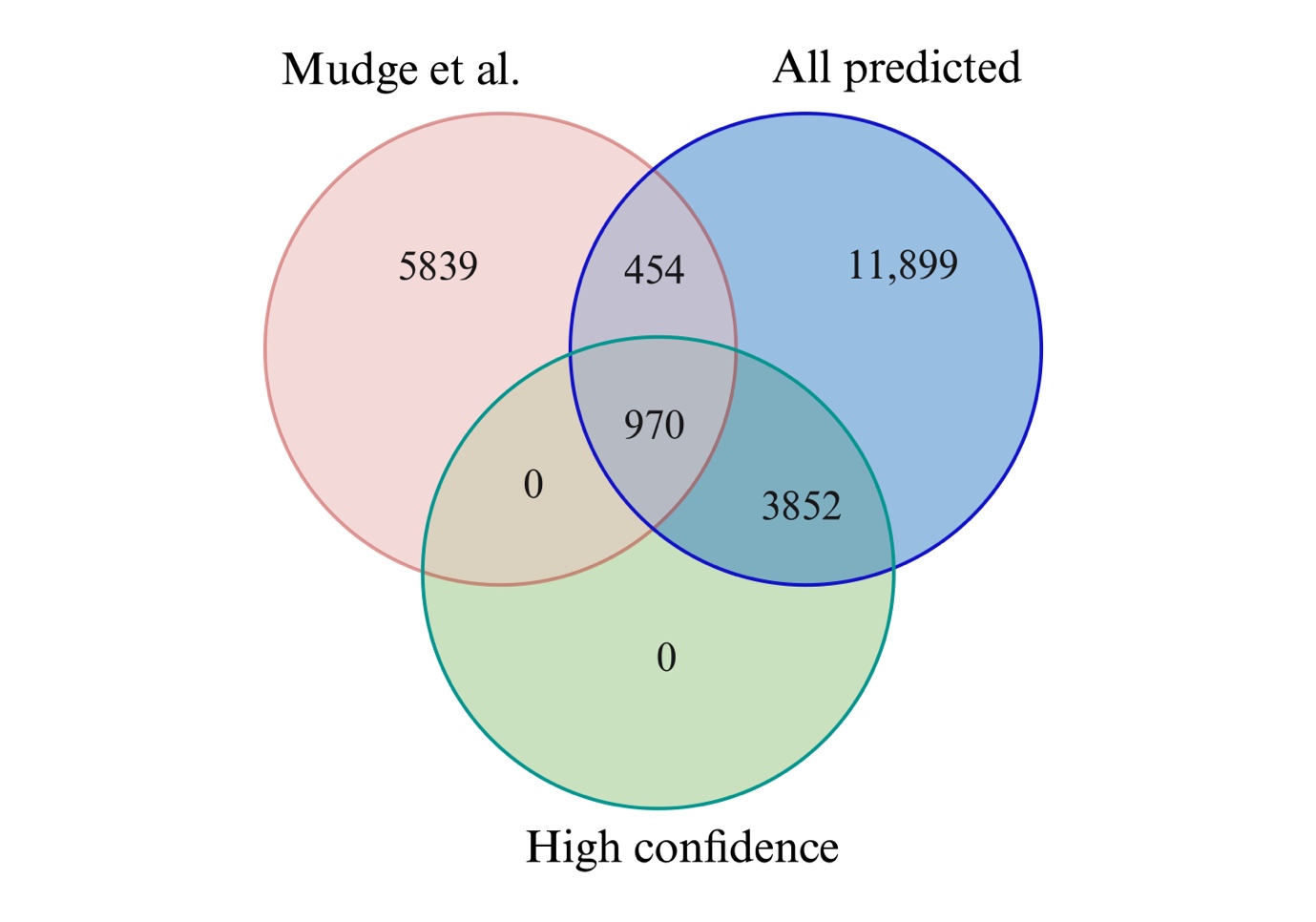
Figure SS9 Overlap of predicted protein coding ORFs with curated set of noncanonical ORFs provided by Mudge et al. in 2022.**

**Conserved ORFs and their recurrent detection across samples**

We observed conserved ORFs are detected more recurrently than those that are poorly conserved.


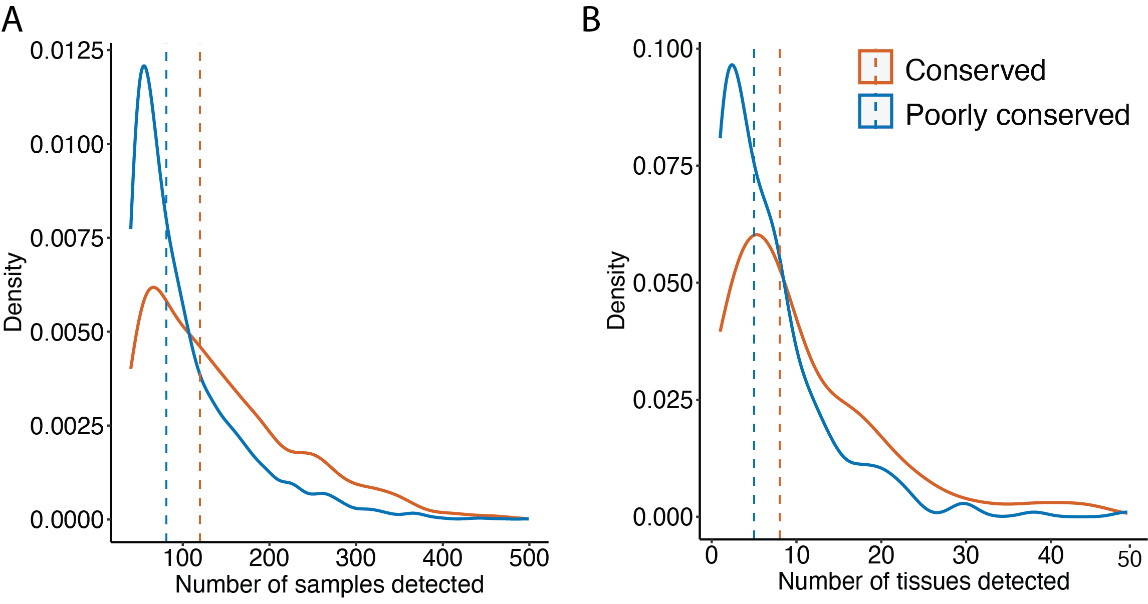


**Figure SS10 Conserved and poorly conserved ORFs detected across samples/tissues.**

**A.** Distribution of conserved and poorly conserved sORFs detected by Ribo-seq. **B**. Distribution of conserved and poorly conserved SEPs detected in proteomics.

**Conservation in predicted candidates compared to undetected candidates**


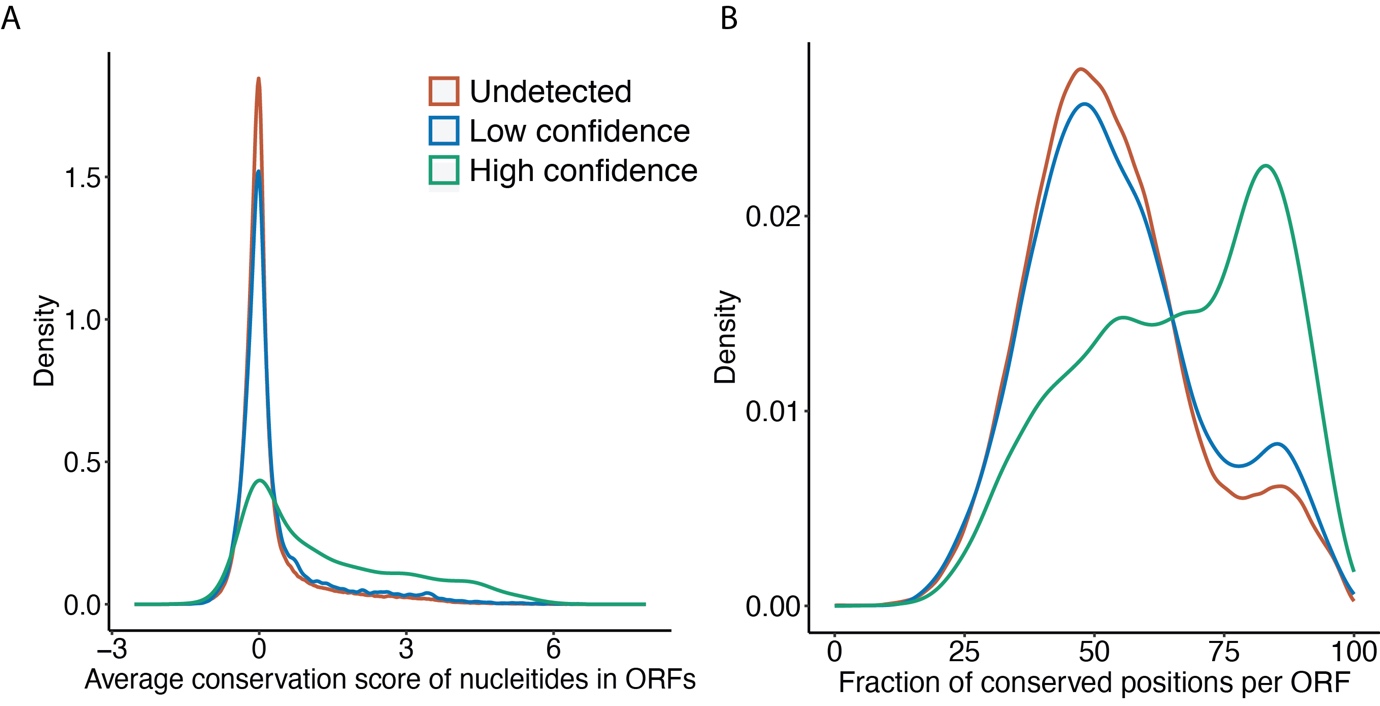


**Figure SS11 Conservation in predicted low and high confidence ORFs compared to undetected candidates.**

**A**. Average conservation in predicted low confidence, high confidence, and undetected candidates. **B**. Fraction of conserved nucleotides in predicted low confidence, high confidence, and undetected candidates.

**Mutation analysis**

Breakdown of high confidence ORFs that showed overlap with mutation coordinates.

**Table SS4 Count of predicted high confidence sORFs that showed overlap with mutation coordinates**

| **Mapping type** | **Ribo-seq** | **Proteomics** | **Both** | **Total** |
| --- | --- | --- | --- | --- |
| SNP-based | 104 | 5 | 4 | 105 |
| Region-based | 318 | 19 | 13 | 324 |

*Note*: SNP, single nucleotide polymorphism.

We considered mutations in CDS regions of newly predicted and unpredicted ORFs. Most of the mutations reside in unpredicted candidates (Figure SS12A). Distribution of mutations within the predicted high confidence candidates shows several of them to be missense, nonsense, and frame-shift mutations, indicating these mutations affecting protein sequence (Figure SS12B).


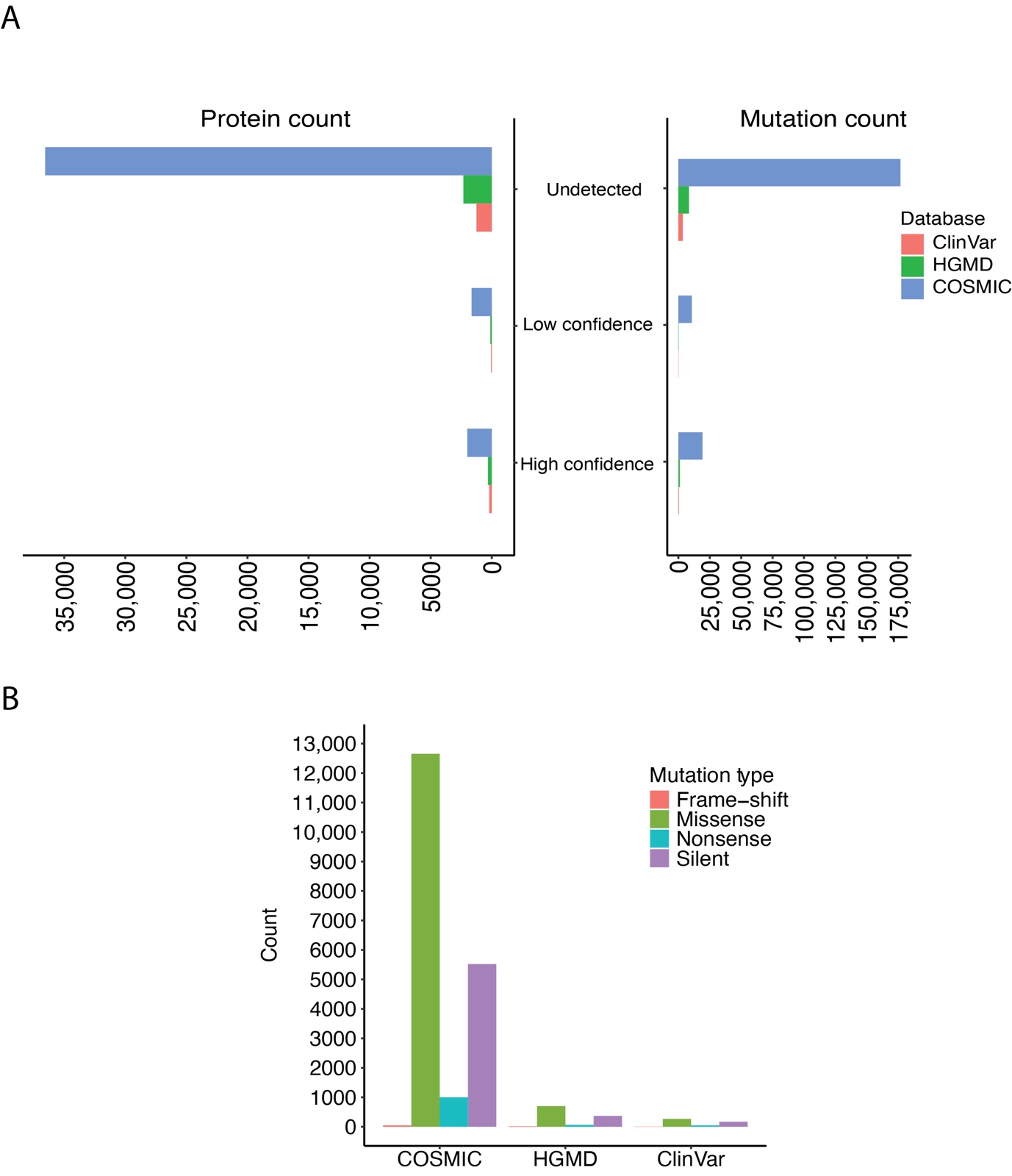


**Figure SS12 Mutations in coding regions of sORFs.**

**A**. Number of sORFs overlap with disease associated mutations from HGMD, ClinVar, and COSMIC. **B**. Distribution of mutations in CDS regions of predicted high confidence candidates according to their mutation types. HGMD, Human Gene Mutation Database; COSMIC, Catalogue of Somatic Mutations in Cancer.

**Disordered residue content in SEPs**

**
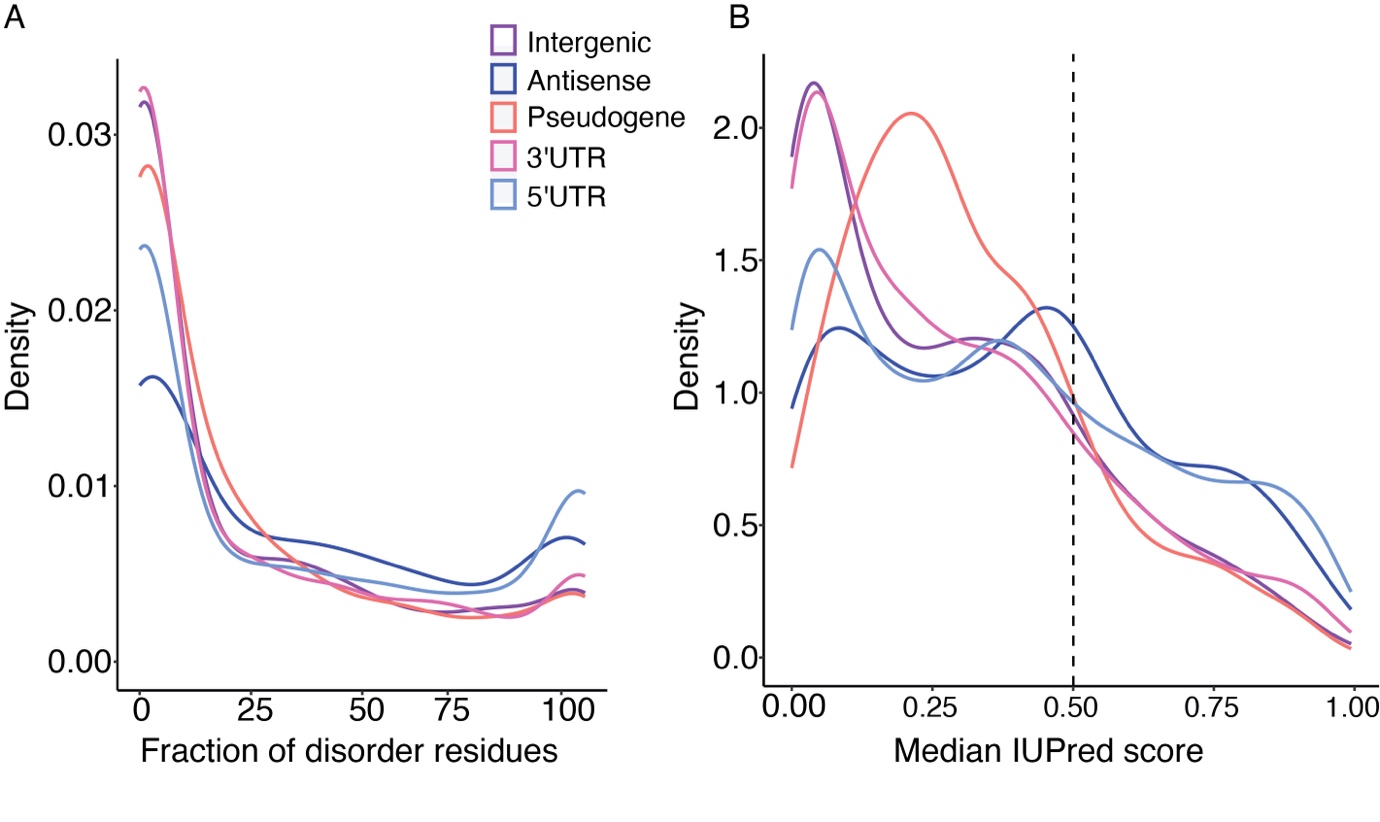
**

**Figure SS13 Disordered residue content in predicted SEPs.**

**A**. Proportion of disordered residues content in predicted SEPs. Residues with IUPred score at least 0.5 were considered disorder. **B**. Median IUPred score in each predicted SEPs. High confidence sORFs were considered in the analysis.
