# Supplementary figures and images for: Identification of Small Open Reading Frame-encoded Proteins in the Human Genome
Source: Genomics Proteomics Bioinformatics. 2025 Feb 7;23(1):qzaf004. doi: 10.1093/gpbjnl/qzaf004 (PMC12236067; doi:10.1093/gpbjnl/qzaf004)

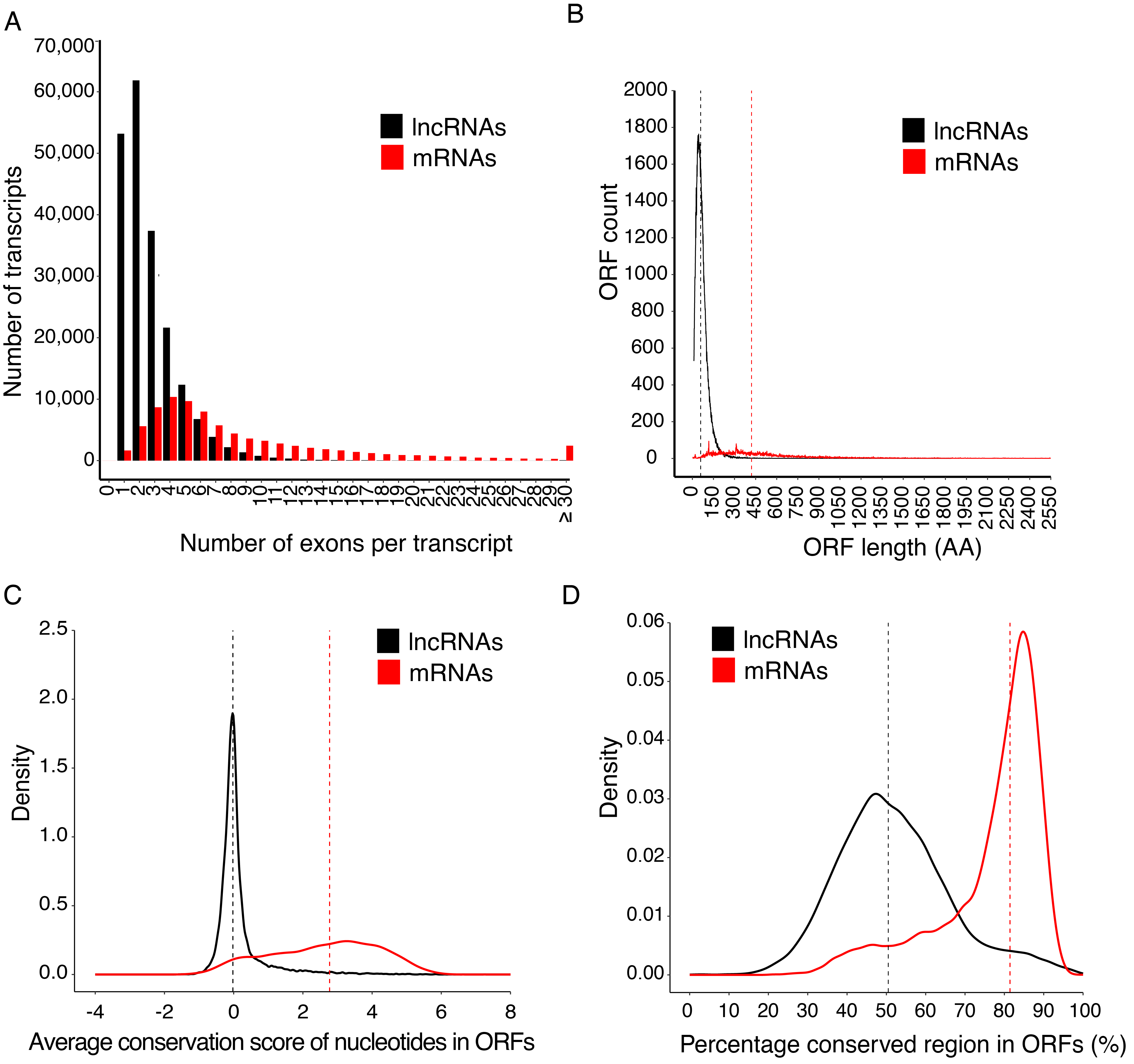

Supplement: qzaf004_Supplementary_Data [file qzaf004_supplementary_data.zip › Figure S1.jpg]

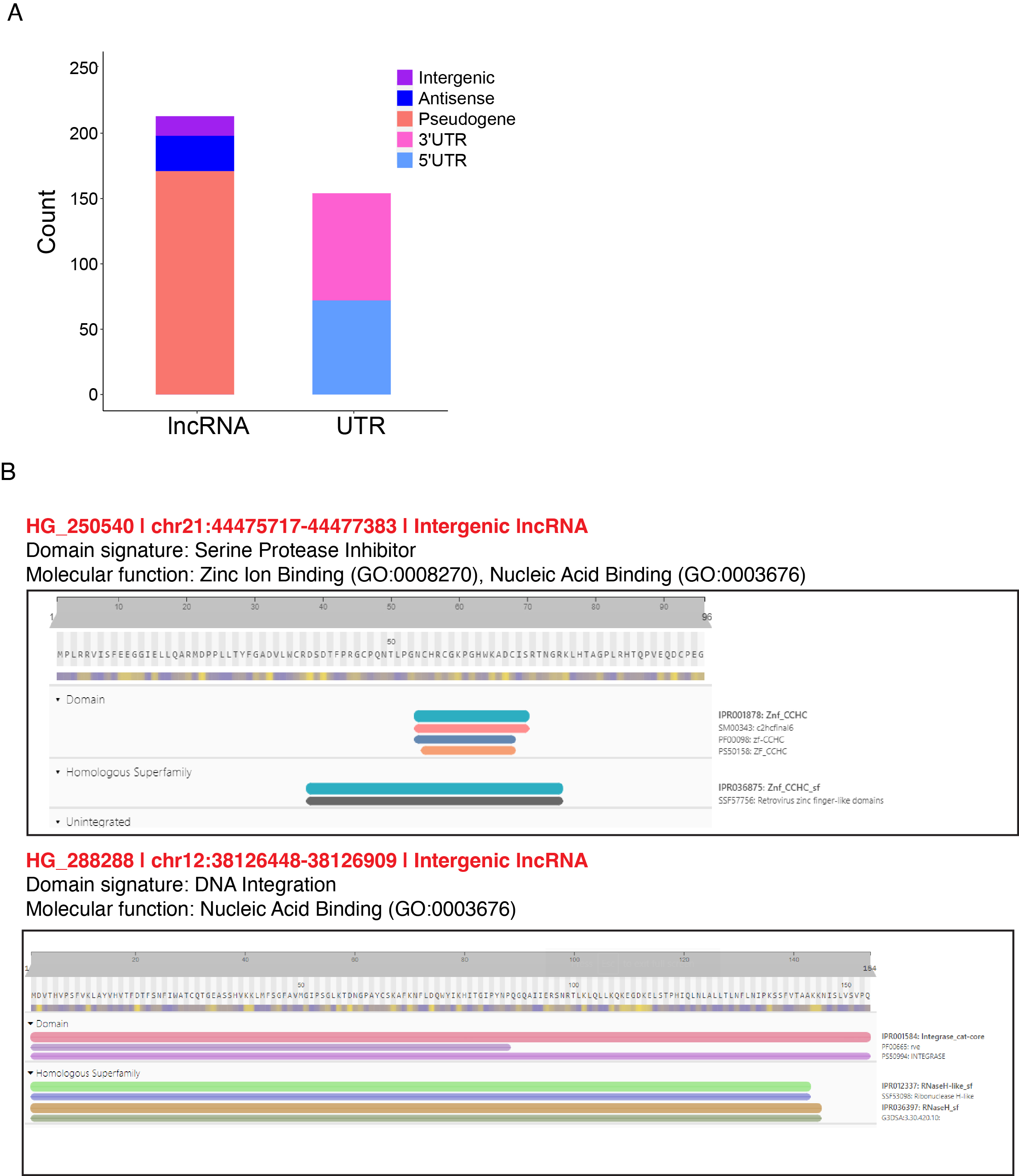

Supplement: qzaf004_Supplementary_Data [file qzaf004_supplementary_data.zip › Figure S8.jpg]

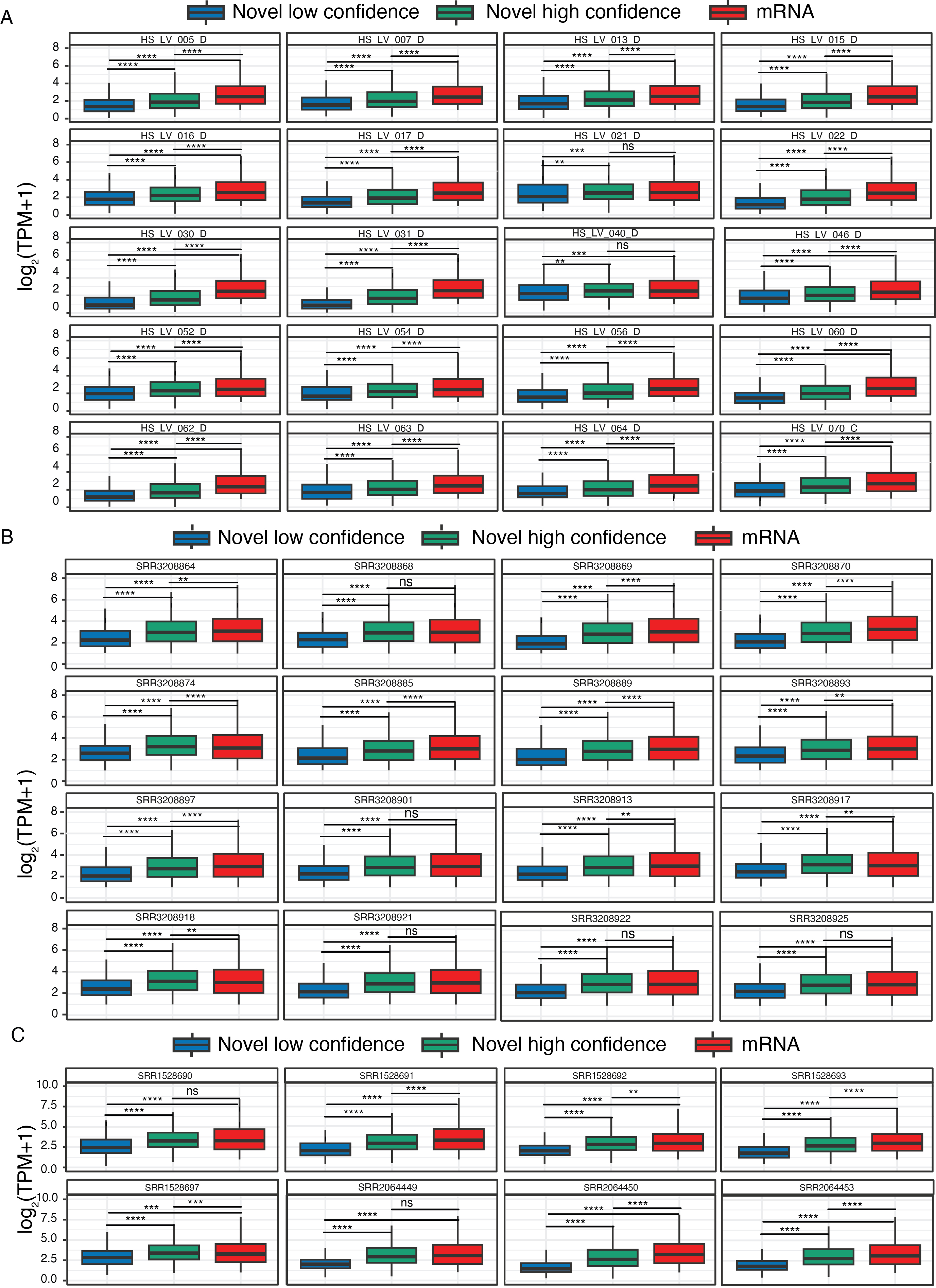

Supplement: qzaf004_Supplementary_Data [file qzaf004_supplementary_data.zip › Figure S4.jpg]

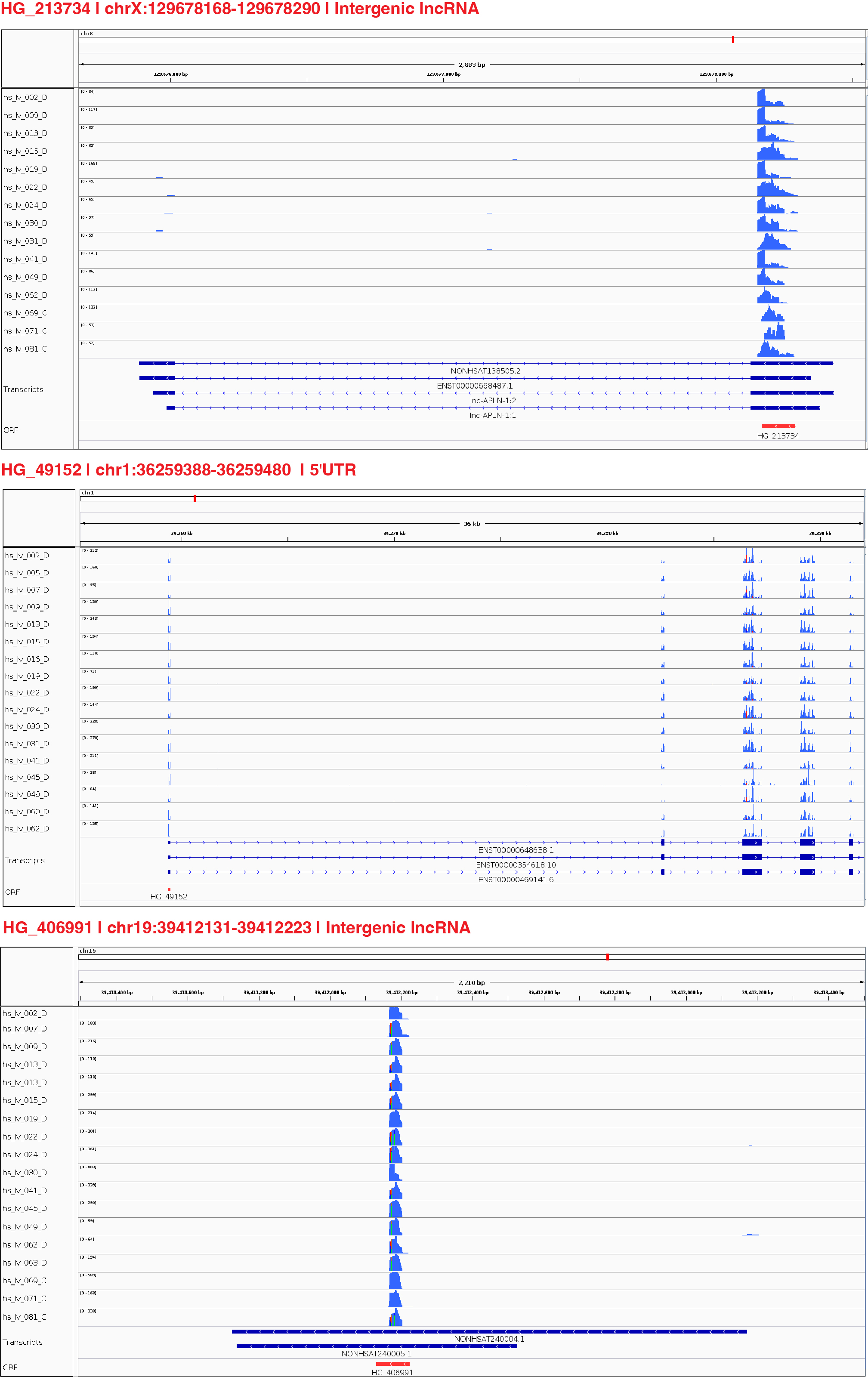

Supplement: qzaf004_Supplementary_Data [file qzaf004_supplementary_data.zip › Figure S5.jpg]

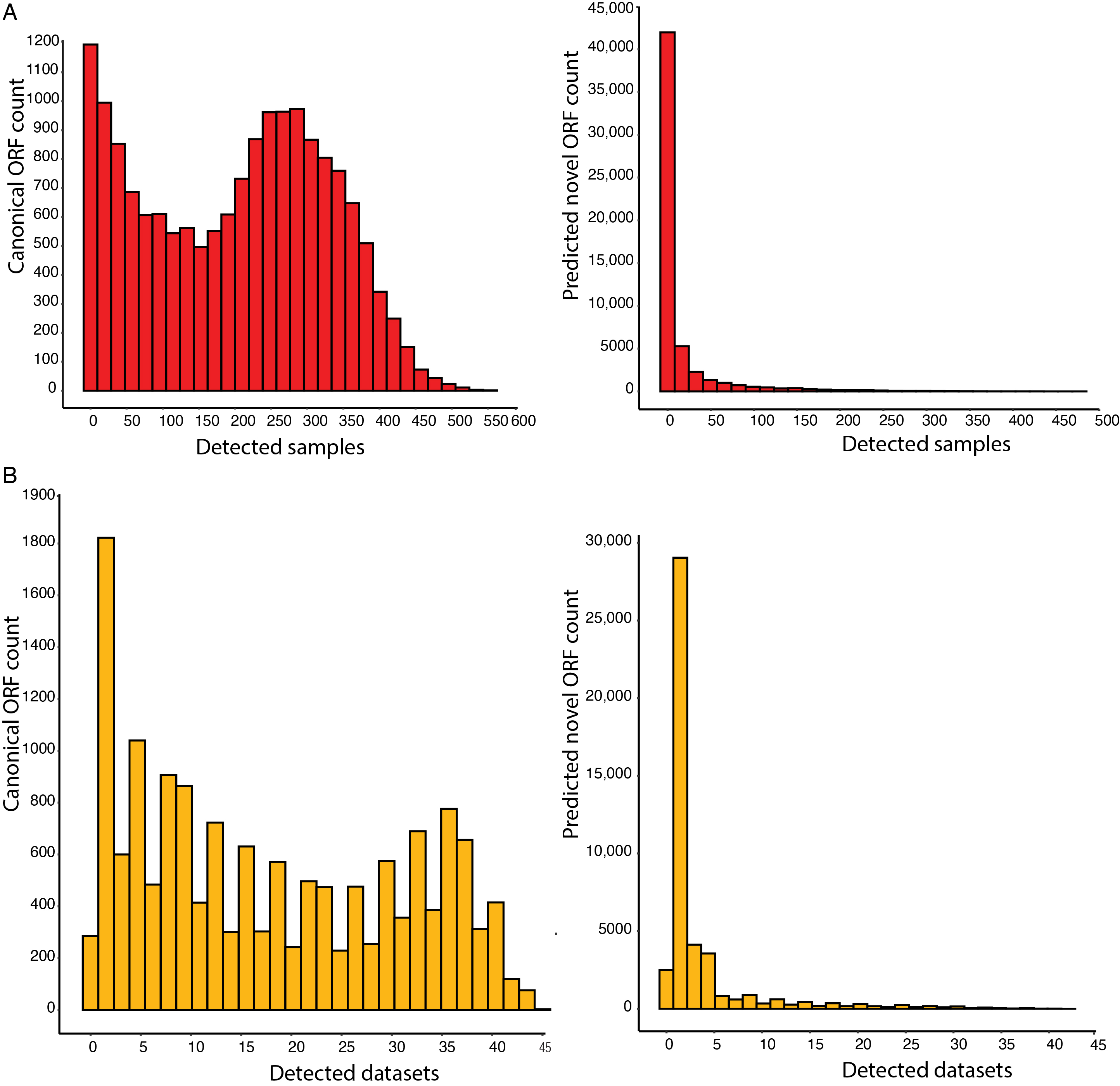

Supplement: qzaf004_Supplementary_Data [file qzaf004_supplementary_data.zip › Figure S3.jpg]

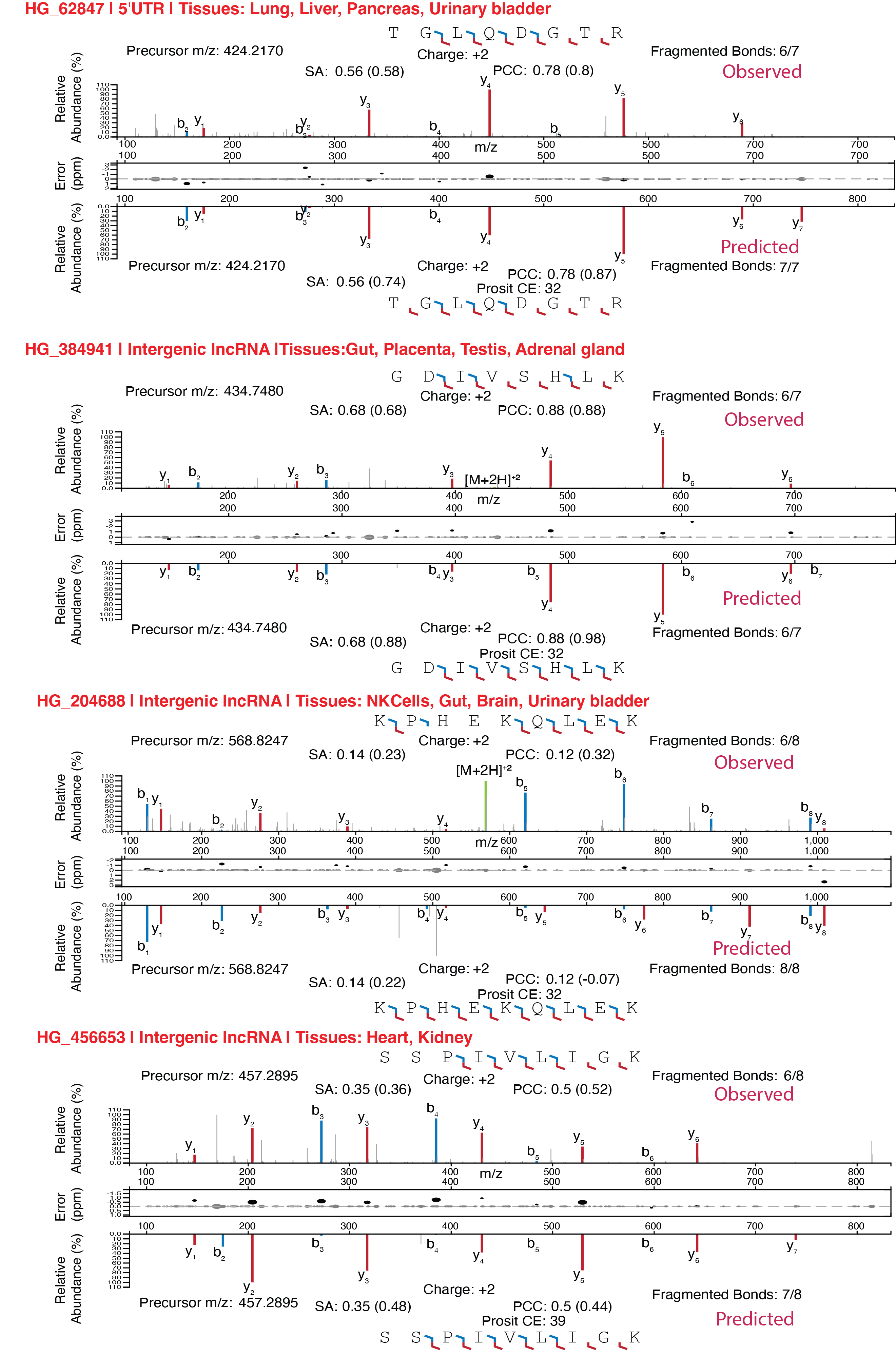

Supplement: qzaf004_Supplementary_Data [file qzaf004_supplementary_data.zip › Figure S6.jpg]

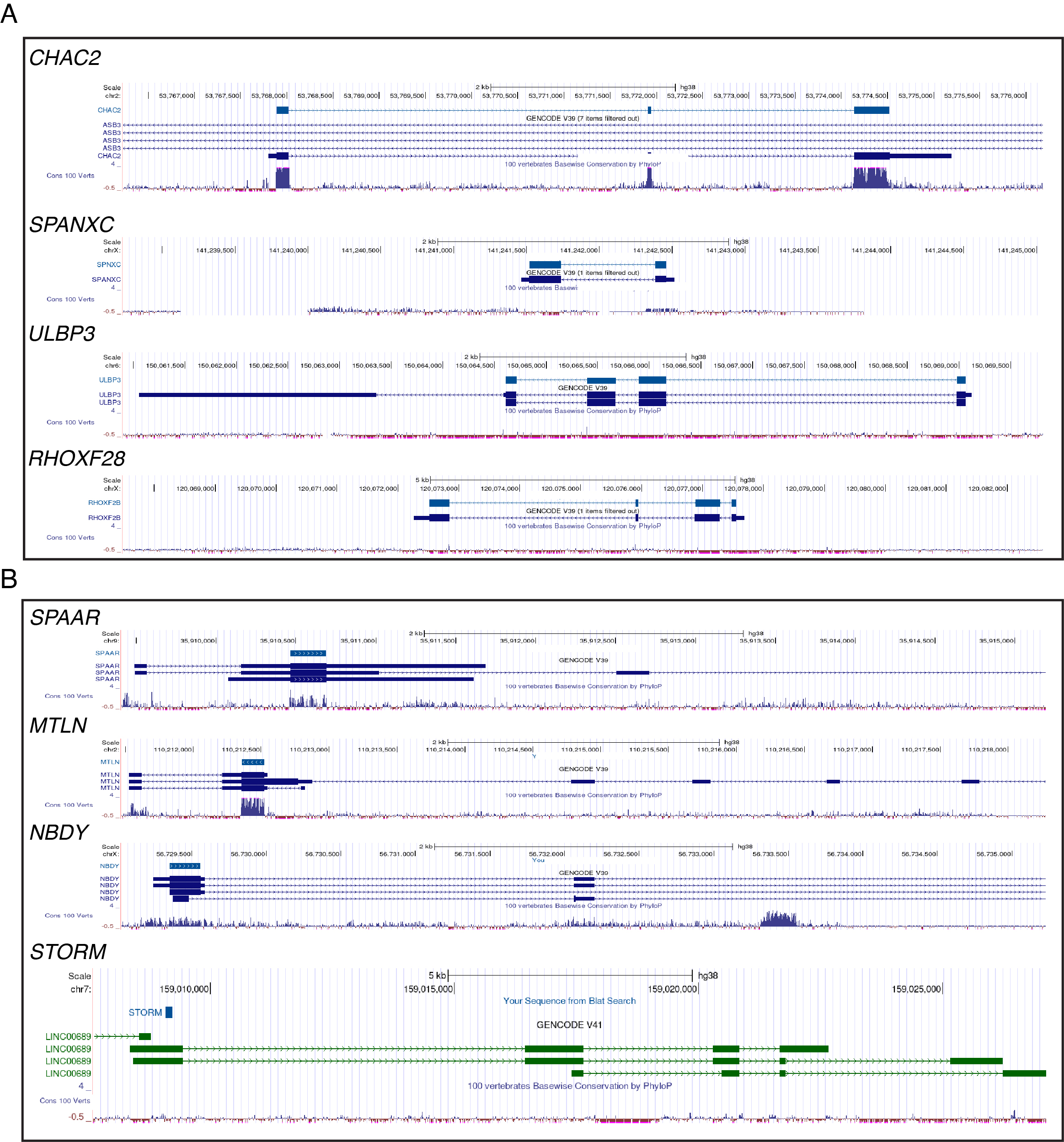

Supplement: qzaf004_Supplementary_Data [file qzaf004_supplementary_data.zip › Figure S2.jpg]

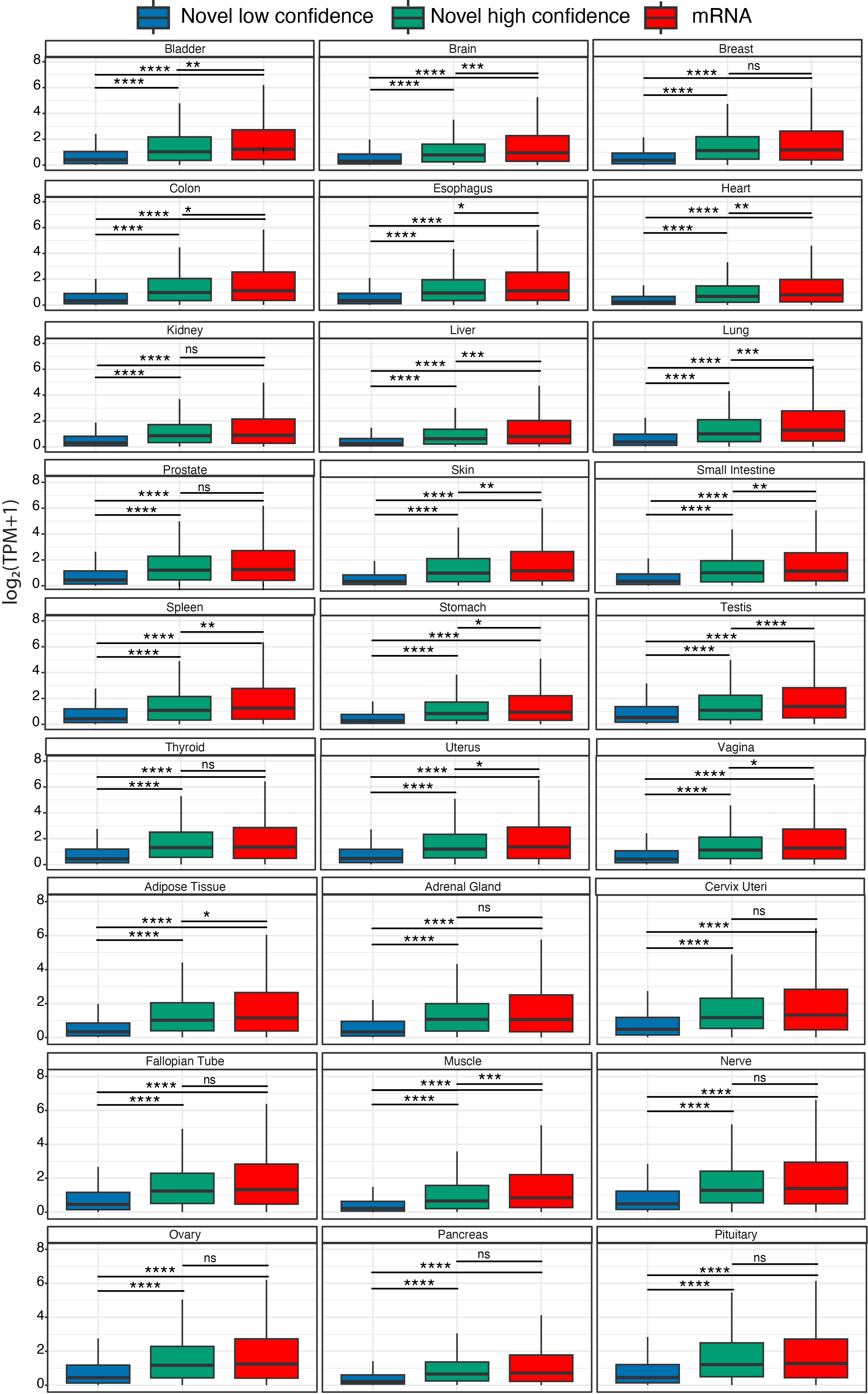

Supplement: qzaf004_Supplementary_Data [file qzaf004_supplementary_data.zip › Figure S7.jpg]
